# Bacterial Community Profiling of Tropical Freshwaters in Bangladesh

**DOI:** 10.3389/fpubh.2019.00115

**Published:** 2019-05-31

**Authors:** Nafisa Azmuda, Md. Fakruddin, Sirajul Islam Khan, Nils-Kåre Birkeland

**Affiliations:** ^1^Department of Microbiology, University of Dhaka, Dhaka, Bangladesh; ^2^Department of Biological Sciences, University of Bergen, Bergen, Norway; ^3^Department of Microbiology, Jahangirnagar University, Savar, Dhaka, Bangladesh

**Keywords:** freshwater lakes, aquatic bacterial community, tropical freshwater, *E. coli-Shigella* group, diarrheagenic *E. coli*, *Shigella*, PCR-DGGE, seasonal variation

## Abstract

Seasonal and spatial variations in the bacterial communities of two tropical freshwater sources in Bangladesh, Lake Dhanmondi in central Dhaka, and a pond in the outskirts of Dhaka, were assessed and compared using PCR-DGGE and deep sequencing of 16S rRNA genes, as well as heterotrophic enrichments using water samples collected at nine different time points during 1 year. Temporal and spatial variations of common aquatic bacterial genera were observed, but no clear seasonal variations could be depicted. The major bacterial genera identified from these two sites were members of the *Proteobacteria, Cyanobacteria, Actinobacteria, Bacteroidetes, Chlorobi, Chloroflexi, Verrucomicrobia*, and *Firmicutes*. Among the proteobacterial groups, members of the *α-*, *β-*, and *γ- Proteobacteria* predominated. *γ- Proteobacteria* belonging to the *Escherichia coli/Shigella* group even the diarrheagenic pathotypes of *E. coli* e.g., EPEC and ETEC were detected in most samples throughout the year, with no apparent correlations with other microbial groups. The other pathotypes, EHEC, EAEC, and EIEC/*Shigella* spp. were also detected occasionally. This study represents the first thorough analysis of the microbial diversity of tropical freshwater systems in Bangladesh.

## Introduction

Microorganisms in freshwater ecosystems play major roles in nutrient cycling and transformation of chemical elements (1). Lakes and other inland waters are early indicators of both regional and global environmental changes (2, 3). Until two decades ago, knowledge of bacteria that naturally occur in freshwater ecosystems was limited to only culturable organisms (4), although it was estimated that about 99% of the bacteria in the environment remains non-culturable when conventional cultural methods are used (5). Molecular cultivation-independent tools have now opened the door to the diversity and composition of bacterial communities in freshwater ecosystems and have revealed distinct and unique bacterial communities among aquatic environments (6, 7). Most of the fingerprinting tools include cultivation-independent approaches for bacterial identification on the basis of sequence variations in variable regions of the 16S rRNA gene. The most frequently used techniques include Terminal Restriction Fragment Length Polymorphism (T-RFLP), Denaturing Gradient Gel Electrophoresis (DGGE), construction of clone 16S rRNA gene libraries, and tagged pyrosequencing (8). All these approaches are based on Polymerase Chain Reaction (PCR) amplification of parts or nearly complete 16S rRNA genes and are as such subject to methodological biases.

The major bacterial divisions so far identified in most or all freshwater sites that have been analyzed are the *Proteobacteria* (*α*, *β*, and *γ* subdivisions), the *Cytophaga*-*Flavobacterium*-*Bacteroides* (CFB) group, the *Actinobacteria*, the *Cyanobacteria*, and the *Verrucomicrobia* (4, 9). However, biological, physical and chemical features play significant roles in the variation of total microbial communities in freshwater lakes. Along with the major permanent members, 16 phyla; the *Acidobacteria, Chlorobi, Cloroflexi*. *Fibrobacteres, Firmicutes, Fusobacteria, Gemmatimonadetes, Lentisphaerae, Nitrospira, OD1, OP10, Planctomycetes, Spirochaetes*, and the candidate phyla BRC1, LD11, SR1, and TM7 were detected occasionally in freshwater lakes (10, 11). Along with the total community profiling, seasonal variations of bacterial community structures have been already observed in different aquatic systems e.g., marine, estuarine, and freshwater ecosystems (12–14). Temperature is one of the most important abiotic factors that is responsible for temporal as well as spatial variations of bacterial community structures (15). There are many reports on marine and soil bacterial communities in tropical regions (9, 16), but very few on tropical freshwater bacterial communities (17).

Along with the autochthonous bacterial community members, tropical environments (e.g., soil and water) also support the survival of foreign microorganisms including enteric bacteria e.g., *Escherichia coli* and *Salmonella entrica* (18). Tropical environments containing high and stable temperatures, high light intensities, and high humidity, in comparison with temperate ecosytems, are drivers for ecological activity and diversity. These abiotic factors play important roles in the survival and adaptation of microbial (pathogens and non-pathogens) species also in a foreign environment (19). The impacts of these factors on bacterial diversity among natural aquatic bacterial communities, as well as allochontous pathogens, were previously proven (20, 21).

The increasing survival potentiality of allochthonous microbiota, especially the pathogens in tropical ecosystems, increases the incidence of waterborne diseases to human communities. Waterborne diseases are one of the major causes of death worldwide including Bangladesh. According to WHO, more than 5 million people die each year because of water-associated diseases (22). Wastewater discharges in fresh waters and coastal seawaters are major sources of fecal pathogens (22, 23). Emerging pathogens that cause waterborne diseases include mainly viruses (Calici and other viruses), bacteria (pathogenic *Escherichia coli, Yersinia, Legionella*, and *Aeromonas* spp.) and protozoa (*Cryptosporidium, Giardia, Entamoeba histolytica*). Besides these “emerging” pathogens, most of the “classical” pathogens are still a matter of concern. The “classical” water-related pathogens include *Vibrio, Salmonella*, and *Shigella* species and hepatitis A virus (24), many of which are enteric pathogens and responsible for causing diarrheal diseases. In Bangladesh, fecal contamination of surface water bodies causes a high prevalence of diarrheal diseases especially in children <5 years of age. The prevalence of diarrhea reported in Bangladesh was 16 per 1,000 persons among all ages and 44 per 1,000 persons in young children (25). In 2015, 6% of 0.119 million death incidences has occured in Bangladesh due to diarrheal diseases (26). Although there are many evidences for the survival of these enteric pathogens in tropical aquatic environments, their survival strategy, association with other aquatic micro- and macroorganism and the mode of transmission are still not well understood. Hence, bacterial community structure analysis of the tropical aquatic environments as in Bangladesh could shed light on understanding of the survival pattern and possible adaptations of these organisms in such foreign environments.

The present study focuses on the total bacterial community profiles with special emphasis on the *E. coli-Shigella* group and their seasonal fluctuations in two freshwater ecosystems (a lake and a pond in Dhaka city and in a rural place in the Brahmanbaria district, Bangladesh) by cultivation-independent molecular approaches, like PCR-DGGE and deep sequencing of 16S rRNA gene amplicons and detection of the five diarrheagenic pathovers of the *E. coli* e.g., Enteropathogenic *E. coli* (EPEC), Enterotoxigenic *E. coli* (ETEC), Enterohemorrhagic *E. coli* (EHEC), Entero aggregative *E. coli* (EAEC) and Enteroinvasive *E. coli* (EIEC), and the *Shigella* species by detection of the virulence marker genes by Multiplex PCR assay. Determination of the total bacterial community structure in tropical freshwater ecosystems like Bangladesh might be helpful for better understanding of the distribution and survival pattern of different bacterial genera in the tropical environment, which could be useful for making comparison with the aquatic bacterial communities in other regions. Seasonal variations in bacterial community structures will help to understand whether the physicochemical factors like temperature, pH, salinity, etc. influence the distribution of the microbial populations and survival pattern of enteric pathogenic bacteria in aquatic ecosystems.

## Materials and Methods

### Collection of Fresh Water Samples

Three sampling sites from two fresh water systems, a pond in Brahmanbaria (N 23°58.874′, E 91°06.607′) and Lake Dhanmondi in Dhaka [site I (N 23°44.854′, E 90°22.724′) and site II (N 23°44.627′, E 90°22.748′)], Bangladesh were selected as the sampling sites. Surface water samples were collected 16 times during July 2008–June 2010 with 1–2 months intervals. Samples for DNA extraction were collected in sterile sampling bottles and were carried to the laboratory on ice. Various physicochemical parameters like temperature, pH, salinity, total dissolved solids (TDS), and conductivity of the water samples were measured by a combined meter (HACH, USA) at the sampling sites.

### Extraction of Total Genomic DNA

About 500 ml water sample were filtered through a 0.45 μm filter (Milipore) and the filter was soaked in Tris-EDTA (TE) buffer (10 mM Tris-HCl, 1.0 mM EDTA, pH 8.0) for 3–4 h. Bacterial cells were then collected from the TE buffer by centrifugation at 12,000 rpm for 10 min and the supernatant was drained off. Genomic DNA was extracted according to a procedure described previously (27).

### Pre-enrichment and Enrichment Techniques for Bacterial Cell Recovery

Fifty ml pre-enrichment medium (0.025% sodium pyruvate, 0.2% proteose peptone, 0.1% K_2_HPO_4_, 0.05% KH_2_PO_4_, 0.3% NaCl, and 10 μg/ml of each of the nucleotide bases; adenine, guanine, thymine, cytosine, and uracil) adjusted to pH 7.5 was used for the recovery of bacterial cells. The subsequent enrichment step was carried out with the same medium supplemented with streptomycin to 50 μg/ml. Fifty ml of water samples were added to 50 ml of 2× (double strength) pre-enrichment medium and incubated for 8–10 h at 37°C in a rotary shaker at 120 rpm. Five ml of samples from the pre-enrichment broth was then transferred to 45 ml of enrichment medium and the incubation was continued for 6 h. A 0.5× (half strength) Nutrient Broth (Oxoid, UK) was used for recovery of the total heterotrophic bacterial populations. Fifty ml of water sample was added to 50 ml of 1× Nutrient Broth and incubated for 16 h under conditions as described for the pre-enrichments. The recovered bacterial cells were harvested after growth in these media by centrifugation at 12,000 rpm for 10 min. Genomic DNA was extracted from each sample according to a procedure described previously (27).

### DGGE Analyses of 16S rRNA Gene Fragments

One hundred and eight DNA samples collected during July 2008 to July 2009 from the three sites were selected for bacterial community structure analysis by DGGE. PCR amplification of the highly variable V1–V4 regions of the bacterial 16S rRNA gene was carried out using the universal bacterial primers, A8-28GCF (28) and K517R (29). This universal primer set amplifies a fragment of 528 bp, which corresponds to position 8 to position 536 in the 16S rDNA of *E. coli*. PCR amplification was performed in a thermal cycler (PTC100, Bio-Rad, USA) in a total volume of 50 μl reaction mixture containing template DNA, 25 μM of each of the primers, 200 μM of the deoxyribonucleotide triphosphate (dNTP) mix, 1 unit of DNA polymerase (DyNazyme^TM^, Finnzymes), and sterile water to make a total volume of 50 μl. The PCR program was initiated with denaturation of template DNA at 94°C for 3 min followed by 30 cycles of the following steps: denaturation at 94°C for 30 s, annealing at 55°C for 30 s, and extension at 72°C for 1 min with a final extension at 72°C for 10 min. DGGE analyses of the PCR amplicons was carried out with 0.75 mm thick polyacrylamide gels and the used denaturing gradient (urea/formamide) was 30–60% (29). Electrophoresis was carried out in a tank (Scie-Plas) containing 5 liters 0.5× TAE (40 mM Tris, 40 mM acetic acid, 1 mM EDTA, pH 7.4) buffer at 60°C for 16 h at 70 V. The gel was stained with SYBR Gold nucleic acid gel stain (0.5 μg/ml, Molecular Probes) for 1 h and visualized under UV illumination (Bio-Rad). DNA bands were excised from the gel and DNA was allowed to diffuse into 20 μl MilliQ water for overnight at 4°C. Five μl of the 20 times diluted elutates were used as templates for reamplification with the same primer set of the 16S rRNA gene. The PCR products were purified and sequenced on an ABI 3700 PE (Applied Biosystems) using the Bigdye terminator kit version 3.1 for automated DNA sequencing. The homology of the 16S rRNA gene sequences was compared with sequences of other organisms that had already been submitted to GenBank using the BLASTN (http://www.ncbi.nih.gov/BLAST/) algorithm (30) and the sequence data were submitted to the GenBank under the accession number-MK271399-MK271632. DGGE band sequences with no or low identity with 16S rRNA gene sequences (<90%) were excluded from the analaysis.

### Deep Sequencing of the 16S rRNA Gene Fragments

Two DNA samples from two sites (Brahmanbaria pond and Lake Dhanmondi site I) were chosen for pyrosequencing according to a previously described procedure (10). Two subsequent PCR reactions, first with untagged primers, followed by a second PCR with the same primers tagged with a 4-base tag and extended with a 5′ 30-nucleotide adaptor, were carried out. The same universal bacterial primers used for DGGE, but without the GC clamp, were used in the first PCR reaction. PCR amplification was performed in a total volume of 50 μl containing 20 ng template DNA, 25 μM of each of the primers, 200 μM of the deoxynucleoside triphosphate (dNTP) mix, 2 mM MgCl_2_, 1 unit of HotStar *Taq* DNA polymerase (Qiagen) and sterile water to make up a total volume of 50 μl. The PCR program was initiated with denaturation of template DNA and HotStar *Taq* DNA polymerase activation at 96°C for 15 min, followed by 20 cycles of the following steps: denaturation at 96°C for 1 min, annealing at 50°C for 1 min, and extension at 72°C for 1 min. A single final extension was done at 72°C for 10 min. The PCR products were purified using the “Sigma GenElute PCR Clean-Up Kit” (Sigma, USA).

The second PCR was done following the similar procedure described above except for using 2.5 mM MgCl_2_ and 100 ng of the purified PCR amplicons as template DNA. The identical primers A8-28F and K517R but with the following added extensions to the 5′ ends: 5′- CCATCTCATCCCTGCGTGTCTCCGACTCAGACAG-3′ and 5′-CCTATCCCCTGTGTGCCTTGGCAGTCTCAG-3′, respectively, were used. The underlined bases in the forward primer extension were used as a tag. The rest of the extensions are adaptor sequences. The PCR program was initiated with denaturation of template DNA at 96°C for 15 min, followed by 2 cycles of the following steps: denaturation at 96°C for 1 min, annealing at 50°C for 1 min, and extension at 72°C for 1 min. The next 8 cycles were: denaturation at 96°C for 1 min, annealing at 65°C for 1 min, and extension at 72°C for 1 min. A single final extension was done at 72°C for 10 min. The PCR products were purified using the “Agencourt AMPure purification kit” (Beckman Coulter). DNA concentration was measured using the nanodrop technique. The sequencing of the amplicons was done commercially at GATC Biotec AG in Germany (http://www.gatc-biotech.com/en/home.html) by the GS FLX (Roche/454) system and Titanium chemistry. The raw sequence data were analyzed using the CLC genomic workbench version 11.

### Multiplex PCR Assay for Detection of *E. coli* and *Shigella* Specific Virulence Genes

Detection of different virulence markers of diarrheagenic *E. coli* and *Shigella* species was performed by multiplex PCR assay using nine set of primer pairs (Table S1) as described previously (31–33). A total of 98 DNA samples (from pre-enrichment and enrichment cultures) collected during July 2008–June 2010 from the three sites were selected for this analysis. The presence of the pathogens were verified as follows: the presence of *eltB* and/or *estA* (enterotoxins of ETEC) for ETEC, the presence of *bfpA* (structural gene for the bundle-forming pilus of EPEC) and *eaeA* (structural gene for intimin of EHEC and EPEC) for typical EPEC (but the presence of only *eaeA* for atypical EPEC), the presence of *vt1* and/or *vt2* (Shiga toxins 1 and 2 of EHEC) for EHEC (additional presence of *eaeA* confirmed the presence of a typical EHEC), the presence of *ial* (invasion-associated locus of the invasion plasmid found in EIEC and *Shigella*) and/or *ipaH* (invasion plasmid antigen found in EIEC and *Shigella*) for EIEC/*Shigella* and the presence of the *Eco*RI*-Pst*I DNA fragment of plasmid pCVD432 of EAEC strains for EAEC. Three different sets of multiplex PCR reactions were carried out depending on the amplicon size (Table S1). PCR was performed in a 30 μl reaction mixture containing 3 μl of template DNA, 3 μl of 10× buffers for DyNAzyme, 0.6 μl of mixture of deoxynucleoside triphosphates (25 mM each), 0.3 μl of 2 U/μl of DyNAzyme^TM^ DNA polymerase (Finnzymes), and 1.0 μl of a 25 μM solution of each primer (Sigma, Germany). Thermocycling conditions were as follows: 94°C for 1 min, 55°C for 1 min, and 72°C for 1 min for 30 cycles, with an initial denaturation at 96°C for 4 min and a final 10-min extension at 72°C. PCR products (10 μl) were evaluated by electrophoresis as described before with a molecular marker (1-kb DNA ladder, Fermentus, UK) concurrently.

## Results

### Physicochemical Parameters of Waterbodies

In Bangladesh there are mainly three seasons–summer (March–May), rainy season (June–September/ early October) and winter (end of October–February). The water temperature ranged between 17 and 23°C in the winter to 31°C in the summer (Tables 1–3). The pH values in Lake Dhanmondi were stable throughout the year, varying between 6.5 and 7.3. In the Brahmanbaria pond the pH ranged from 6.0 in July 2008 to 8.0 in November/December 2008 (Table 1). The salinity of Lake Dhanmondi and Brahmanbaria pond was detected as 0.1 and 0.2‰, respectively which was stable throughout the whole year. The range of TDS and conductivity value were 104–188 mg/l and 209–450 μS/cm for Lake Dhanmondi. However, in Brahmanbaria pond the TDS values were 187–266 mg/l and conductivity values were 383–512 μS/cm throughout the year (Figures S1–S3).

**Table 1:** Distribution of bacterial genera in Brahmanbaria Pond in July 2008–2009.

**Taxon**	**Brahmanbaria Pond**								
	**July 2008**	**Sept. 2008**	**Nov. 2008**	**Dec. 2008**	**Jan. 2009**	**Feb. 2009**	**April 2009**	**June 2009**	**July 2009**
Temperature (°C)	30.0	30.0	27.0	23.0	23.8	27.6	28.0	31.0	31.0
pH	6.0	7.0	8.0	8.0	7.0	7.0	7.5	7.5	7.0
*Aeromonas*	^b^P^c^E^d^T	PET	^a^DPT	PT	PT	T	DPT	PT	PT
*Acinetobacter*	P		E		PT	T	P		
*Bacillus*					E		E		PE
*Comamonas*			PE	E	DPET	DT	PET	DPET	DPET
*Cyanobium*	E	D	D	DE	D	D	D	D	D
*Escherichia coli/Shigella*	E	DP	DE	PE	ET		E		
*Exiguobacterium*					PT	T	PET		
*Kurthia*	T	T	T		T	T	PET	DT	
*Microcystis*							D	DPET	
*Vogesella*					DPET	DT	DPET	DPET	PET

a *D, Direct DNA extract from water*;

b *P, DNA extract from pre-enrichment culture*;

c E, DNA extract from enrichment culture, and

d *T, DNA extract from nutrient broth culture*.

**Table 2:** Distribution of bacterial genera in Lake Dhanmondi site I in July 2008–2009.

**Taxon**	**Lake Dhanmondi site I, Dhaka**								
	**July 2008**	**Sept. 2008**	**Nov. 2008**	**Dec. 2008**	**Feb. 2009**	**Mar. 2009**	**April 2009**	**June 2009**	**July 2009**
Temperature (°C)	29.8	30.2	25.8	20.0	20.0	30.5	30.0	29.0	31.0
pH	7.0	7.0	7.0	7.0	7.0	7.0	7.0	7.0	7.0
*Aeromonas*	PET	^b^PT		PE	PET	DPE		PE	T
*Acinetobacter*	ET		PT						T
*Bacillus*			DT	ET	ET	DP	E	E	E
*Burkholderiales*		^a^D							
Candidatus *Pelagibacter*	D								D
*Comamonas*	ET	ET	DPET	PET	E	PE	E	E	E
*Cyanobium*	D			D	DPET	DP	E	DP	D
*Enterobacter*		^c^E							
*Escherichia coli / Shigella*	ET	E			E	DE			
*Exiguobacterium*						E			
*Kurthia*		^d^T							
*Lysinibacillus*						E			
*Methylophilus*		D							
*Moraxella*			DPE						
*Plesiomonas*									E
*Polynucleobacter*						D			
*Pseudomonas*	DPET							T	E
*Vogesella*	DPT	T	DPT	PET	DET	DPT	ET	PT	DPT

a *D, Direct DNA extract from water*;

b *P, DNA extract from pre-enrichment culture*;

c E, DNA extract from enrichment culture, and

d *T, DNA extract from nutrient broth culture*.

**Table 3:** Distribution of bacterial genera in Lake Dhanmondi site II in July 2008–2009.

**Taxon**	**Lake Dhanmondi site II, Dhaka**								
	**July 2008**	**Sept. 2008**	**Nov. 2008**	**Dec. 2008**	**Feb. 2009**	**Mar. 2009**	**April 2009**	**June 2009**	**July 2009**
Temperature (°C)	29.5	30.0	25.6	20.0	20.0	30.5	31.0	29.0	31.0
pH	7.0	7.0	7.0	7.0	7.0	7.0	7.0	7.0	7.0
*Aeromonas*	DPT	PT	^b^P	PET	PT	PET	P	P	DP
*Aquabacterium*	^a^D		P	PT					
*Bacillus*						PET	^c^E		
*Bacterium*	D								
Candidatus *Pelagibacter*	D		D					DE	D
*Comamonas*	DE	PE	PE	PE	P		E	PET	PET
*Cyanobium*	DE		D	D	DP		D	DP	D
*Enterobacter*		E							
*Escherichia coli/Shigella*	PET	P	DE	DE	DP				
*Exiguobacterium*			P						
*Kurthia*					^d^T	P			
*Lysinibacillus*						T			E
*Methylobacter*		D							
*Methyloversatilis*		D							
*Panacagrimonas*		DE							
*Plesiomonas*			E		P				
*Polynucleobacter*							D		
*Vogesella*	DPET	P	DPT	PT	PT	PET	PT	DPET	DPT

a *D, Direct DNA extract from water*;

b *P, DNA extract from pre-enrichment culture*;

c E, DNA extract from enrichment culture, and

d *T, DNA extract from nutrient broth culture*.

### Bacterial Community Structure Analysis by PCR-DGGE

Analysis of the total bacterial community in two freshwater ecosystems of Bangladesh along with seasonal variations was carried out by the separation of 16S rRNA gene PCR amplicons by DGGE followed by sequencing. The DGGE profiles of the collected water samples from the two water reservoirs (three sites) and in different months demonstrated a diverse composition of the bacterial communities with representatives of several indigenous freshwater bacterial genera, like *Aeromonas, Acinetobacter, Bacillus, Comamonas, Cyanobium, Kurthia, Vogesella* as well as representatives of enteric bacteria like the *E. coli*-*Shigella* group. A number of bands containing sequences of high similarity with still uncultured bacterial groups were also determined.

**Figure 1:**
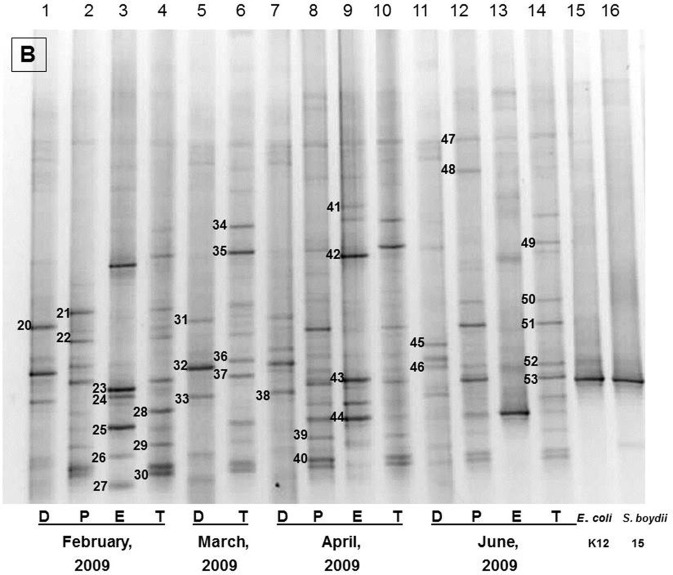
The representative DGGE profiles of bacterial community of Brahmanbaria pond in February 2009–July 2009. D, P, E, and T stand for the Direct, Pre-enriched, Enriched, and Total heterotrophic DNA samples.

**Figure 2:**
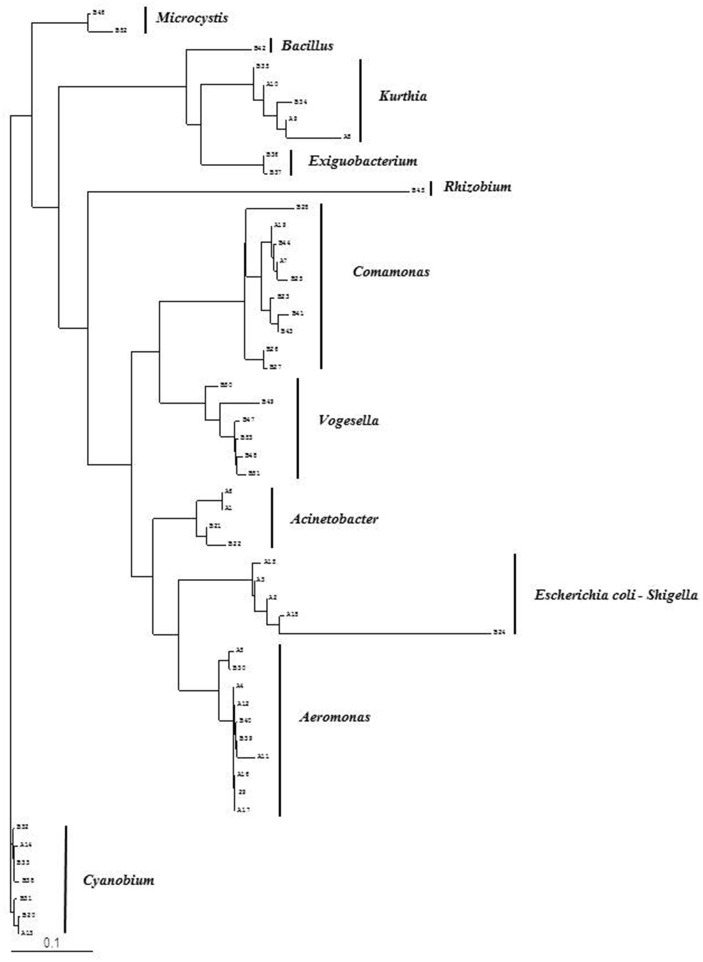
Phylogenetic diversity of bacterial community in Brahmanbaria pond based on DGGE (2008–2009). The tree has been rooted with *Cyanobium* sp.

**Figure 3:**
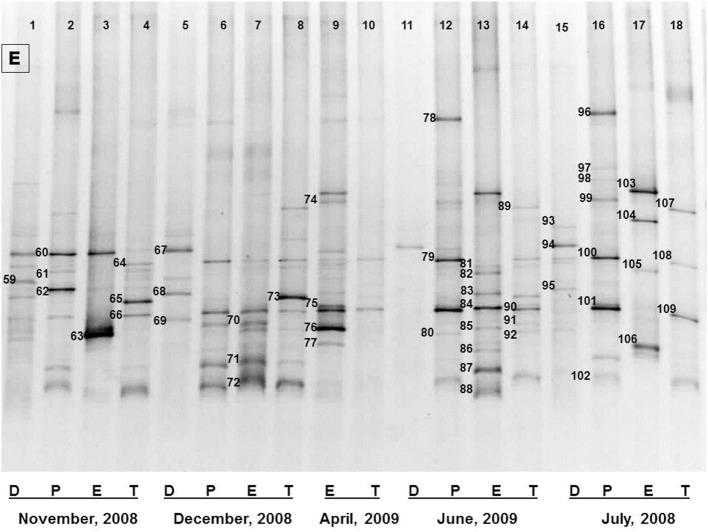
The representative DGGE profiles of bacterial community of Dhanmondi Lake site I in July 2008–June 2009. D, P, E, and T stand for the Direct, Pre-enriched, Enriched, and Total heterotrophic DNA samples.

**Figure 4:**
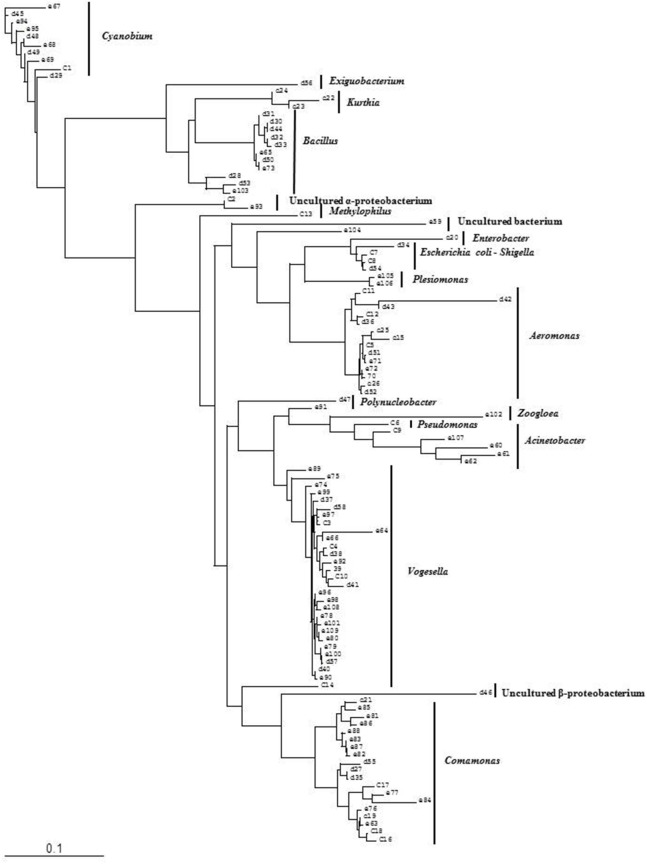
Phylogenetic diversity of bacterial community in Lake Dhanmondi, Dhaka site I based on DGGE (2008–2009). The tree has been rooted with *Cyanobium* sp.

**Figure 5:**
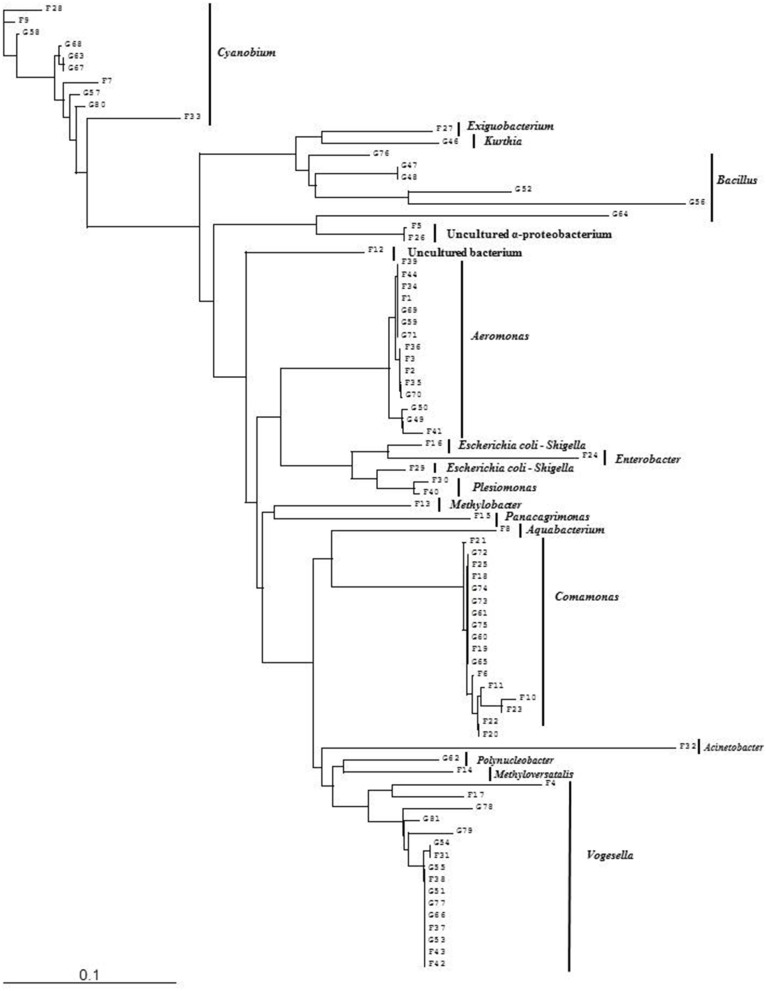
Phylogenetic diversity of bacterial community in Lake Dhanmondi, Dhaka site II based on DGGE (2008–2009). The tree has been rooted with *Cyanobium* sp.

Bacterial species and genera identified in the three sites throughout the year, along with the temperature and pH fluctuations are indicated in Tables 1–3. The DGGE profiles of the collected water samples from these sites revealed the presence of highly diverse bacterial communities with stable and transient members. The stable members included the representatives of major common and indigenous freshwater bacterial genera, like *Acinetobacter, Aeromonas, Bacillus, Comamonas, Cyanobium* (and other cyanobacteria), *Kurthia, Vogesella* as well as representatives of enteric bacteria-the *E. coli*-*Shigella* group (Tables 1–3,  Tables S2–S4, Figures 1–5, Figures S4–S7) among the sites. Various transient members responsible for spatial variations within these communities included the member of the genera *Acetobacter, Aquabacterium*, *Burkholderiales*, *Enterobacter*, *Exiguobacterium, Methylobacter*, *Methyloversatilis*, *Moraxella*, *Panacagrimonas*, *Plesiomonas*, *Polynucleobacter*, and *Zooglea*. The Brahmanbaria pond maintained a more stable but different bacterial community in comparison to Lake Dhanmondi which had uncommon stable members like *Exiguobacterium*, with only one transient member *Microcystis*. A number of unidentified or “uncultured” bacteria were also occasionally detected, which were distantly related to the genera *Bacterium*, Candidatus genus *Pelagibacter, Ramlibacter, Steroidobacter*, etc. (Tables 1–3). These organisms shared from 71 to 92% 16S rRNA sequence identity with their closest cultivated relatives (Tables S2–S4) and were most often detected in direct DNA extracts, indicating the lack of growth under laboratory culturing conditions (Figures S4–S7).

However, no clear seasonal variations were observed among the identified stable members within these aquatic systems. Most of these bacteria were recovered by cultural means but with few exceptions. The *E. coli-Shigella* group was mostly detected in pre-enrichment and enrichment DNA extracts, but for a few direct DNA extracts from Lake Dhanmondi site II and Brahmanbaria pond, a positive PCR reaction was obtained, indicating a heavy load of enteric bacteria at the respective times. Except for being undetected in June/July 2009 in all the sites, there was no systematic seasonal fluctuation in the presence or absence of this group.

### Bacterial Community Structure Analysis Determined by Deep Sequencing

**Figure 6:**
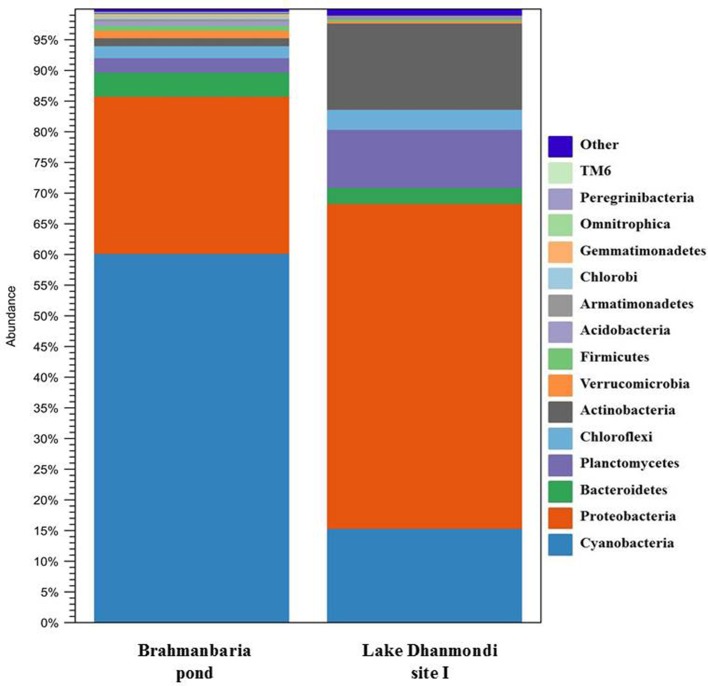
Diversity of bacterial phyla in Brahmanbaria pond and Lake Dhanmondi site I in November 2008 and July 2008, respectively based on Deep 16S rDNA sequencing.

**Figure 7:**
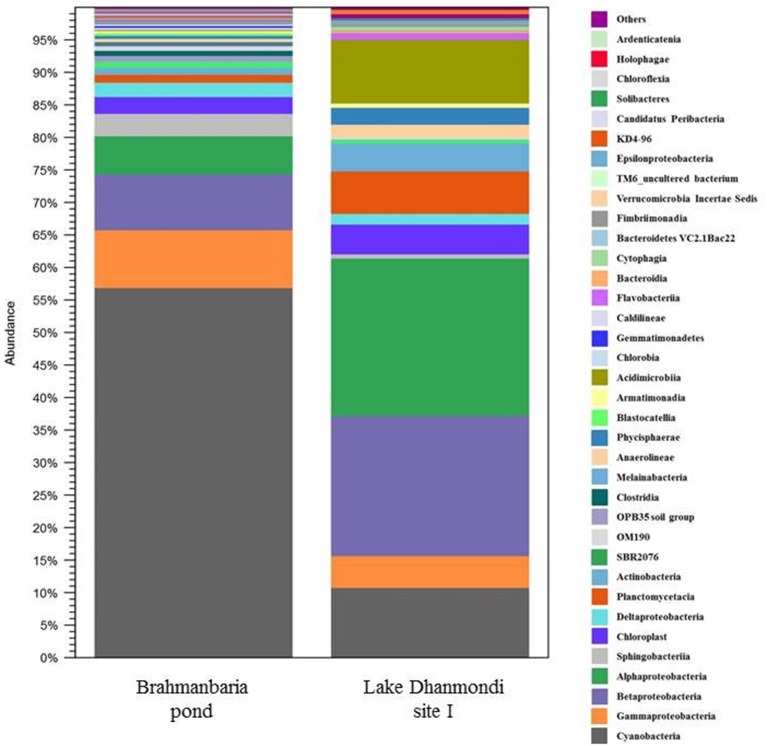
Distribution of bacterial classes in Brahmanbaria pond and Lake Dhanmondi Site I, based on deep 16S rDNA sequencing.

Deep sequencing of 16S rRNA gene amplicons based on two direct DNA extracts from Lake Dhanmondi site I (July 2008), and Brahmanbaria pond (November 2008) was done for in-depth analysis of the bacterial community structure in these two sites and also to investigate whether this method could directly detect possible enteropathogens without any pre-enrichment or enrichment steps. For Lake Dhanmondi sample, the total reads in OTUs were 292 and number of OTUs matched with database were 78. For Brahmanbaria pond, the total OTU reads were 18,011 and number of matched OTUs with database were 496. The major bacterial phyla identified from these two sites were: *Cyanobacteria, Proteobacteria, Bacteroidetes, Planctomycetes, Chloroflexi, Actinobacteria, Verrucomicrobia, Firmicute, Acidobacteria, Armatimonadetes, Chlorobi, Gemmatimonadetes*, etc. (Figures 6, 7) but with spatial variations. *Cyanobacteria* was the most dominant phylum in the Brahmanbaria pond, with 60% of the total number of reads, while Lake Dhanmondi was dominated by *Proteobacteria* and *Actinobacteria* and with *Cyanobacteria* constituting about 15% of the community (Figure 6). A large number of bacterial classes and genera within these phyla were identified (Figure 6), representing diversified bacterial communities in both the waterbodies. This indicates that the Brahmanbaria pond is a typical natural freshwater environment where photoautotrophs are the primary energy producers while the Lake Dhanmondi is dominated by heterotrophs as a result of a heavy load of organic material. In both the sites, the dominant *Proteobacteria* were the *α-*, *β-*, and *γ- Proteobacteria* comprising 6, 8, and 8% respectively of the total bacterial population of Brahmanbaria pond whereas it compromised 24, 21, and 5%, respectively of the total bacterial population of Lake Dhanmondi (Figure 6). Among the *γ- Proteobacteria*, the *Enterobacteriaceae* member the *E. coli-Shigella* group was detected in Brahmanbaria pond but in low numbers.

### Detection of Diarrheagenic *E. coli* Pathotypes and *Shigella* spp.

Identification of diarrheagenic pathotypes of *E. coli* and *Shigella* spp. was carried out by multiplex PCR assays for Brahmanbaria pond and Lake Dhanmondi with samples obtained during two consecutive years (July 2008 - June 2010). For these analyses, DNA extracts from pre-enrichment and enrichment cultures were used. The diarrheagenic pathotypes of *E. coli* identified in these sites are summarized in Table 4, based on the presence of specific virulence marker genes as shown in Table S5. Among the dirrheagenic pathotypes of *E. coli*, EPEC followed by ETEC were frequently identified in all the sites throughout the whole year with occasional absence. EIEC and/or *Shigella* spp. were detected mostly in November to February (winter season) showing seasonal as well as spatial variation. The most pathogenic type EHEC and another pathotype EAEC was detected occasionally in the Brahmanbaria pond whereas those were absent in Lake Dhanmondi. No clear seasonal variation of the pathotypes was observed, indicating that the distribution is due to a more or less continuous fecal discharge of these organisms.

**Table 4:** Presence of diarrheagenic *E. coli* pathotypes and *Shigella* spp. in Brahmanbaria pond and Lake Dhanmondi, Dhaka in July 2008–June 2010.

**Sampling sites**	**Year**	**Months**									
		**July**	**Sept**.	**Oct**.	**Nov**.	**Dec**.	**Jan**	**Feb**.	**March**	**April**	**June**
Brahman-baria Pond	2008–2009	EPEC, ^e^EAEC	^b^ETEC, ^c^EPEC	^a^ND	ETEC, EPEC, EIEC/*Shigella* sp.		EPEC		ND	EHEC	EPEC
	2009–2010	EPEC	ETEC	ND	^d^EHEC, EIEC/ *Shigella* sp.		^f^EIEC/ *Shigella* sp.		ND		
Lake Dhanmondi site I, Dhaka	2008–2009			ND	EIEC/ *Shigella* sp.	ETEC	ND			EPEC	
	2009–2010			ND	EPEC		ND	EIEC/ *Shigella* sp.		EPEC	EPEC
Lake Dhanmondi site II, Dhaka	2008–2009	ETEC, EPEC		ND	EPEC		ND			ETEC	EPEC
	2009–2010	ETEC		ND		ETEC	ND	ETEC	EPEC	ETEC, EPEC	EPEC

a *ND, Not done*;

b *ETEC, Enterotoxigentic E. coli*;

c *EPEC, Enteropathogenic E. coli*,

d *EHEC, Enterohemorrhagic E. coli*;

e *EAEC, Enteroaggregative E. coli*;

f *EIEC, Enteroinvasive E. coli*.

## Discussion

The PCR-DGGE approach combined with deep sequencing of 16S rRNA gene amplicons was used to study the total bacterial community structure along with the distribution and seasonal fluctuations of the bacterial genera in tropical freshwater ecosystems in Bangladesh. In addition to direct molecular analysis of water samples, pre-enrichment and enrichment cultures were also assessed in order to enhance the recovery of fastidious bacteria from environmental waters. The pre-enrichment medium was designed to resuscitate stressed or dormant bacterial cells and the enrichment medium was used mainly for the recovery of *Shigella* species where streptomycin created the selective pressure (34). Another, non-selective medium with diluted nutrient concentration (0.5× Nutrient broth) was used for the recovery of total heterotrophs.

Recently, several studies on the bacterial community composition in the epilimnion of freshwater lakes and reservoirs have been performed. Major autochthonous bacterial populations have been shown to represent at least 7 different phyla (4, 11). Variations on bacterial community composition depends on several factors, such as regionalization, seasonal patterns, and environmental parameters (35, 36). Physiologic predisposition and nutritional tolerance of dominant bacteria tend to maintain stable communities during certain seasons (13). In this study, PCR-DGGE analysis of the 16S rRNA genes was carried out to identify the total bacterial community and seasonal fluctuation on the bacterial populations in two freshwater reservoirs from Dhaka city and Brahmanbaria district. The DGGE profiles revealed the presence of highly diversified bacterial communities in these sites with representatives of major common and indigenous freshwater bacterial genera all through the year and correlates with a previous study (11). Among the two water reservoirs, the Brahmanbaria pond maintained a stable bacterial community structure containing the members of the *β-Proteobacteria*, *γ-Proteobacteria, Cyanobacteria*, and *Firmicutes* as autochothonous members. In Lake Dhanmondi, members of *α-Proteobacteria* were also detected along with the other groups but mostly as transient members. However, in both the sites the diversity and prevelance of *γ-Proteobacteria* was much higher than *β-Proteobacteria*.

The *E. coli-Shigella* group, although not being members of the indigenous aquatic bacterial groups, was frequently detected in all the sites almost throughout the year, with few occasional absences (June/July 2009), but with no clear seasonal patterns. This group was mostly detected in the DNA samples collected after pre-enrichment and enrichment, which proves the efficiency of the media for the recovery of these organisms, as the recovery rate was very low in the nutrient broth (0.5×) due to high competition with other heterotrophs. In a few cases, this group of organisms was detected in “uncultivated” direct water sample extracts, which may indicate the presence of a rather high number of cells. No direct correlation was found for the presence of this group of bacteria with other indigenous or transient bacterial groups detected in these ecosystems.

In this study the seasonal variations among the *E. coli*-*Shigella* group was correlated only with temperature changes although it is known that pH is another strong determinant for bacterial community structures (15). The sites in Lake Dhanmondi, Dhaka maintained a stable neutral pH (~7.0) throughout the year (2008–2009) but it varied in the Brahmanbaria pond (6.0–8.0). A particularly high number of bacterial genera were detected in neutral to alkaline pH in this site. However, the low pH did not seem to affect the survival of the *E. coli-Shigella* group. According to the physical parameters, the lake and pond selected in this study are considered as eutrophic and hypereutrophic because of the presence of high nutrient concentrations, high pH, and thus high conductivity. In shallow eutrophic and hypereutrophic water bodies a dominance of *α-* and *β-Proteobacteria, Bacteroidetes*, and *Actinobacteria* could be found (37–39). However, in the selected sites, *γ*-*Proteobacteria*, especially *E. coli/Shigella* were also a prominent group, supporting the notion of adaptation of this group of bacteria in tropical waters like in Bangladesh. Although the *E. coli-Shigella* group is known as the inhabitants of the human intestine and the lower intestine of other warm-blooded animals, there was evidence of survival of this bacterial group in tropical waters, especially in South-East region (17). Many reports also showed the possibility of multiplication of *E. coli* in tropical waters (40). Although the presence of *Shigella* in tropical environmental waters has been reported but its recovery is extremely difficult following conventional cultural techniques (27, 41). Recent studies have also identified stable genotypes of *E. coli* as “naturalized strains” in the environment, suggesting that certain populations readily circulate and persist outside the host GI tract (42–44). These evidences support the hypothesis that a significant proportion of the global *E. coli* population is not transmitted directly between hosts via the fecal-oral route but instead flows between the environment and hosts (45). Possible adaptation of pathogenic variants of *E. coli* and/or *Shigella* spp. in tropical fresh water environments is going to be a major alarming health concern in this region. Adaptation of non-pathogenic *E. coli* strains in tropical waters also decreases the credibility of using this organism as indicators of fecal contamination.

The Roche/454 next-generation DNA sequencing technology has already been applied to microbial ecology for in-depth analysis of microbial diversity in various ecosystems such as deep mines, soil, deep marine biospheres, chronic wounds, tidal flats, human oral microflora, marine waters, and fresh water reservoirs (16, 46–52). As part of the survey of the total bacterial community structure in freshwater sources of the tropical region, deep 16S rRNA gene amplicon sequencing was done for two direct water sample extracts; from Lake Dhanmondi site I (sampled in July 2008) and Brahmanbaria pond (sampled in November 2008). The major bacterial phyla identified from these two sites were *Cyanobacteria, Proteobacteria, Bacteroidetes, Planctomycetes, Chloroflexi, Actinobacteria, Verrucomicrobia, Firmicute, Acidobacteria, Armatimonadetes, Chlorobi, Gemmatimonadetes*, etc. A large variety of bacterial classes within these phyla were also identified, representing both autochthonous and allochthonous community members. The findings matched previous results from aquatic bacterial community structure analyses from various regions of the ecospheres (4, 11, 51). Lake Dhanmondi is a big reservoir of water in comparison with the Brahmanbaria pond and contains a heavy load of organic material. This explains that the dominant bacterial groups here are the heterotrophs (*Proteobacteria* and *Actinobacteria*); whereas the Brahmanbaria pond represents a more typical natural freshwtaer environment dominated by *Cyanobacteria*, which represent primary energy producers in this ecosystem. Earlier studies showed that among the freshwater aquatic proteobacterial members the *β-Proteobacteria* were most abundant (11). In the current study, a different scenerio was observed in both the water reservoirs with *α-* and *γ- Proteobacteria* as the major proteobacterial groups along with the *β-Proteobacteria* that could be an unique trait for tropical aquatic bacterial communities. Low numbers of *E. coli* or *Shigella* were also detected within the members of *γ- Proteobacteria* in Brahmanbaria pond through this analysis. The absence of this group in Lake Dhanmondi indicated that the number of enteropathogens in this aquatic environment might be too low for detection by this technique using direct DNA extracts, but would probably work for pre-enrichments or selective enrichments.

The molecular analyses successfully fulfilled the primary aim of this study by describing the total bacterial communities as well as detection of the *E. coli-Shigella* group in selected freshwater ecosystems. Additional methods were applied to identify *E. coli* and *Shigella* spp. pathotypes in these aquatic ecosystems. As environmental water plays the major role in spreading diarrheal disease, it is very important to know the distribution and survival patterns of the diarrheal agents in the water bodies. Multiplex PCR assays targetting the virulence genes of diarrheagenic *E. coli* and *Shigella* spp. were carried out to assess their seasonal distribution in the selected freshwater sites for two consequtive years (July 2008–June 2010) using DNA extracted from pre-enrichment and enrichment cultures. Among the diarrheagenic pathotypes of *E. coli*, Enteropathogenic *E. coli* (EPEC) followed by Enterotoxigenic *E. coli* (ETEC) were frequently identified throughout the years in both the sites while the Enteroinvassive *E. coli* (EIEC) and/or *Shigella* spp. were detected mostly in early winter and post winter months. Previously, mostly ST or both ST and LT positive ETEC strains were found prevalent in Bangladesh (53). Later, in a 2% surveillance system at the icddr,b hospital in Dhaka, Bangladesh between 2007 and 2012, the prevalence of ST producing strains had decreased (54). In the present study, the environmental prevalence of ETEC bacteria also correlates with the 6-year surveillance data as the presence of LT toxin gene were higher than only ST or both ST and LT producing genes. In Bangladesh, among all diarrheal pathogens, prevalence of *Shigella* spp. was about 40% in 2008–2009 whereas 25% in 2010 (55). However, the detected EPEC in the selected sites were atypical as only *eaeA* gene was detected (Table S5). The other pathotypes—Enteroaggregative *E. coli* (EAEC) and typical Enterohemorrhegic *E. coli* (EHEC) containing *vt1* and/or *vt2* toxin gene(s) and *eaeA* were rarely detected (only once) each year but only in Brahmanbaria pond hence clarifies a suitable environment for the pathogenic bacteria. This type of distribution of the pathogens might be due to a more or less continual fecal discharge of these organisms by animal or human as the pond in Brahmanbaria was frequently used for human and animal bath. However, no significant correlation between the diarrheagenic *E. coli—Shigella* pathotypes and the aquatic bacterial genera identified by the PCR-DGGE approach was observed in these sites. It was found earlier in Bangladesh that ETEC are the cause of 90% of infant diarrhea and follows a very characteristic biannual seasonality with two separate peaks; in the spring and in the autumn months, but now it remains endemic all the year. EAEC has also been implicated as a cause of sporadic diarrhea in Bangladesh (56). Shigellosis maintains a regular seasonal pattern in Bangladesh with two distinct peaks in pre-monsoon and in the winter period (41). There was only one report on prevelance of diarrheagenic pathotypes of *E. coli* in surface waters in Bangladesh which showed that the ETEC was the most prevalent pathotype, followed by EPEC, and these were distributed throughout the country in three seasons. However, the EIEC and EAEC pathotypes could not be detected in winter and in the rainy seasons, respectively (31).

The combination of PCR-DGGE, deep 16S rDNA sequencing, and detection of virulence genes of diarrheagenic *E. coli* and *Shigella* species, has been successfully applied for the first time to in-depth analysis of freshwater bacterial communities with emphasis on the *E. coli-Shigella* group in a tropical region like Bangladesh. Next generation sequencing is a high-throughput and more quantitative technique for analyzing microbial diversity in comparison with DGGE. The PCR-DGGE approach is cost effective and has been successfully employed previously for the analysis of bacterial communities in surface and ground water systems in Bangladesh (57, 58) and hence the reasons behind application of this technique in the current study for analysis of seasonal variations on bacterial communities. However, through the PCR-DGGE approach seasonal variations on bacterial community structure were only observed in between the dominant bacterial groups. The use of a multiplex PCR assay was much more successful compared to DGGE for detection of the *E. coli/Shigella* group as positive PCR reactions with the virulence gene primers were often obtained for samples that were negative for *E. coli* in the DGGE experiments. Multiplex PCR also provided a far better resolution regarding the specific pathotypes that were present in the samples. Identification of more diversified bacterial populations through deep sequencing approach is the indication of the unavailability of suitable culture media that could be used for the isolation of these bacteria from freshwater ecosystems. The results of the PCR-DGGE approach has also supported the hypothesis, as the pre-enrichment and enrichment media used in this study have recovered many viable but non-culturable (VBNC) cells and supported their growth outside the habitat. Designing of appropriate recovery medium could increase the rate of isolation of many novel aquatic bacterial genera from the environment.

## Conclusion

This study clearly illustrated the total bacterial community profile presented in the tropical aquatic environments like in Bangladesh for the first time. This study also presents evidence of possible adaptation of *γ- Proteobacteria* specially the *E. coli-Shigella* group including their pathotypes in these ecosystems. Culture-independent molecular analyses played a significant role in identification of the total bacterial community along with the seasonal variations. Adaptation of the diarrheagenic pathovers of *E. coli* and *Shigella* species in the aquatic environment is an alarming concern for more frequent occurences of respective diarrheal diseases as environmental water is mostly responsible for spreading the disease in human communities. Long term survival of these groups of bacteria in environmental water also decreases the credibility of *E. coli* of being the indicator bacteria for fecal contamination in tropical regions.

## Author Contributions

NA conceived and designed the research plans, conducted the laboratory works, analyzed and interpreted the data, prepared the manuscript, approved publication of the content, and agreed to be accountable for all aspects of the work. MF collected the water samples and conducted the PCR-DGGE part in laboratory. SK supervised the laboratory work in Bangladesh and also provided scrupulous thoughts on the manuscript. N-KB supervised the whole work and revised the manuscript critically for important intellectual content.

### Conflict of Interest Statement

The authors declare that the research was conducted in the absence of any commercial or financial relationships that could be construed as a potential conflict of interest.

## References

[1] Cotner JB Biddanda BA Small players, large role: microbial influence on biogeochemical processes in pelagic aquatic ecosystems. Ecosystems. (2002) 5:105–21. 10.1007/s10021-001-0059-3

[2] Magnuson JJ Benson BJ Kratz TK Temporal coherence in the limnology of a suite of lakes in Wisconsin, USA. Freshwater Biol. (1990) 23:145–59. 10.1111/j.1365-2427.1990.tb00259.x

[3] Williamson CE Dodds W Kratz TK Palmer MA Lakes and streams as sentinels of environmental change in terrestrial and atmospheric processes. Front. Ecol. Environ. (2008) 6:247–54. 10.1890/070140

[4] Zwart G Crump BC Kamst-van Agterveld MP Hagen F Han S-K Typical freshwater bacteria: an analysis of available 16S rRNA gene sequences from plankton of lakes and river. Aquat Microb Ecol. (2002) 28:141–55. 10.3354/ame028141

[5] Amann RI Ludwig W Schleifer KH. Phylogenetic identification and in situ detection of individual microbial cells without cultivation. Microbiol Rev. (1995) 59:143–69. 7535888 10.1128/mr.59.1.143-169.1995 PMC239358

[6] Rappe MS Vergin K Giovannoni SJ. Phylogenetic comparisons of a coastal bacterioplankton community with its counterparts in open ocean and freshwater systems. FEMS Microbiol Ecol. (2000) 33:219–32. 10.1016/S0168-6496(00)00064-7 11098073

[7] Zwart G van Hannen EJ Kamst-van Agterveld MP Van der Gucht K Lindstrom ES Van Wichelen J. Rapid screening for freshwater bacterial groups by using reverse line blot hybridization. Appl Environ Microbiol. (2003) 69:5875–83. 10.1128/AEM.69.10.5875-5883.2003 14532039 PMC201233

[8] Jansson BPW (2011). The Gut Microbiota: Ecology and Function. Washington, DC: American Soceity for Microbiology.

[9] Ding X Peng XJ Jin BS Xiao M Chen JK Li B. Spatial distribution of bacterial communities driven by multiple environmental factors in a beach wetland of the largest freshwater lake in China. Front Microbiol. (2015) 6:129. 10.3389/fmicb.2015.00129 25767466 PMC4341555

[10] Azmuda N Rahman MZ Madsen MS Khan SI Birkeland NK. Prevalence of a novel division-level bacterial lineage in Lake Dhanmondi, Dhaka, Bangladesh, as revealed by deep sequencing of 16S rRNA gene amplicons. Curr Microbiol. (2012) 65:356–60. 10.1007/s00284-012-0165-9 22706799

[11] Newton RJ Jones SE Eiler A McMahon KD Bertilsson S. A guide to the natural history of freshwater lake bacteria. Microbiol Mol Biol Rev. (2011) 75:14–49. 10.1128/MMBR.00028-10 21372319 PMC3063352

[12] Hofle MG Haas H Dominik K. Seasonal dynamics of bacterioplankton community structure in a eutrophic lake as determined by 5S rRNA analysis. Appl Environ Microbiol. (1999) 65:3164–74. 10388718 10.1128/aem.65.7.3164-3174.1999 PMC91471

[13] Pinhassi J Hagstrom A Seasonal succession in marine bacterioplankton. Aquat Microb Ecol. (2000) 21:245–56. 10.3354/ame021245

[14] Selje N Simon M Composition and dynamics of particle-associated and free-living bacterial communities in the Weser estuary, Germany. Aquat Microb Ecol. (2003) 30:221–37. 10.3354/ame030221

[15] Lindstrom ES Kamst-Van Agterveld MP Zwart G. Distribution of typical freshwater bacterial groups is associated with pH, temperature, and lake water retention time. Appl Environ Microbiol. (2005) 71:8201–6. 10.1128/AEM.71.12.8201-8206.2005 16332803 PMC1317352

[16] Jing H Xia X Suzuki K Liu H. Vertical profiles of bacteria in the tropical and subarctic oceans revealed by pyrosequencing. PLoS ONE. (2013) 8:e79423. 10.1371/journal.pone.0079423 24236132 PMC3827353

[17] Kenzaka T Yamaguchi N Prapagdee B Mikami E Nasu M Bacterial community composition and activity in urban rivers in Thailand and Malaysia. J Health Sci. (2001) 47:353–61. 10.1248/jhs.47.353

[18] Winfield MD Groisman EA. Role of nonhost environments in the lifestyles of Salmonella and Escherichia coli. Appl Environ Microbiol. (2003) 69:3687–94. 10.1128/AEM.69.7.3687-3694.2003 12839733 PMC165204

[19] de Wit R Bouvier T. ‘Everything is everywhere, but, the environment selects’; what did Baas Becking and Beijerinck really say? Environ Microbiol. (2006) 8:755–8. 10.1111/j.1462-2920.2006.01017.x 16584487

[20] Guernier V Hochberg ME Guegan JF. Ecology drives the worldwide distribution of human diseases. PLoS Biol. (2004) 2:e141. 10.1371/journal.pbio.0020141 15208708 PMC423130

[21] Pommier T Canback B Riemann L Bostrom KH Simu K Lundberg P. Global patterns of diversity and community structure in marine bacterioplankton. Mol Ecol. (2007) 16:867–80. 10.1111/j.1365-294X.2006.03189.x 17284217

[22] Fenwick A. Waterborne infectious diseases–could they be consigned to history? Science. (2006) 313:1077–81. 10.1126/science.1127184 16931751

[23] World Health Organization Guidelines for Drinking-Water Quality, Incorporating 1st and 2nd Addenda, Volume 1, Recommendations, 3rd ed. Geneva: WHO (2008).

[24] Egli T Koster W Meile L. Pathogenic microbes in water and food: changes and challenges. FEMS Microbiol Rev. (2002) 26:111–2. 10.1111/j.1574-6976.2002.tb00603.x 12069876

[25] Chowdhury F Khan IA Patel S Siddiq AU Saha NC Khan AI. Diarrheal illness and healthcare seeking behavior among a population at high risk for diarrhea in Dhaka, Bangladesh. PLoS ONE. (2015) 10:e0130105. 10.1371/journal.pone.0130105 26121650 PMC4485467

[26] UNICEF Committing to Child Survival: A Promise Renewed. Progress Report 2015. New York, NY: UNICEF (2015).

[27] Faruque SM Khan R Kamruzzaman M Yamasaki S Ahmad QS Azim T. Isolation of Shigella dysenteriae type 1 and S. *flexneri* strains from surface waters in Bangladesh: comparative molecular analysis of environmental *Shigella* isolates versus clinical strains. Appl Environ Microbiol. (2002) 68:3908–13. 10.1128/AEM.68.8.3908-3913.2002 12147489 PMC124020

[28] Edwards U Rogall T Blocker H Emde M Bottger EC. Isolation and direct complete nucleotide determination of entire genes. Characterization of a gene coding for 16S ribosomal RNA. Nucleic Acids Res. (1989) 17:7843–53. 10.1093/nar/17.19.7843 2798131 PMC334891

[29] Muyzer G de Waal EC Uitterlinden AG. Profiling of complex microbial populations by denaturing gradient gel electrophoresis analysis of polymerase chain reaction-amplified genes coding for 16S rRNA. Appl Environ Microbiol. (1993) 59:695–700. 7683183 10.1128/aem.59.3.695-700.1993 PMC202176

[30] Altschul SF Gish W Miller W Myers EW Lipman DJ. Basic local alignment search tool. J Mol Biol. (1990) 215:403–10. 10.1016/S0022-2836(05)80360-2 2231712

[31] Akter S Islam M Afreen KS Azmuda N Khan SI Birkeland NK. Prevalence and distribution of different diarrhoeagenic Escherichia coli virulotypes in major water bodies in Bangladesh. Epidemiol Infect. (2013) 141:2516–25. 10.1017/S0950268813000320 23445775 PMC9151377

[32] Svenungsson B Lagergren A Ekwall E Evengard B Hedlund KO Karnell A. Enteropathogens in adult patients with diarrhea and healthy control subjects: a 1-year prospective study in a Swedish clinic for infectious diseases. Clin Infect Dis. (2000) 30:770–8. 10.1086/313770 10816147

[33] Venkatesan MM Buysse JM Kopecko DJ. Use of Shigella flexneri ipaC and ipaH gene sequences for the general identification of *Shigella* spp. and enteroinvasive *Escherichia coli*. J Clin Microbiol. (1989) 27:2687–91. 2687318 10.1128/jcm.27.12.2687-2691.1989 PMC267109

[34] Rahman MZ Azmuda N Hossain MJ Sultana M Khan SI Birkeland NK. Recovery and characterization of environmental variants of Shigella flexneri from surface water in Bangladesh. Curr Microbiol. (2011) 63:372–6. 10.1007/s00284-011-9992-3 21826486

[35] Lindstrom ES Leskinen E. Do neighboring lakes share common taxa of bacterioplankton? Comparison of 16S rDNA fingerprints and sequences from three geographic regions. Microb Ecol. (2002) 44:1–9. 10.1007/s00248-002-0007-6 12016460

[36] Yannarell AC Triplett EW. Geographic and environmental sources of variation in lake bacterial community composition. Appl Environ Microbiol. (2005) 71:227–39. 10.1128/AEM.71.1.227-239.2005 15640192 PMC544217

[37] Allgaier M Grossart HP. Diversity and seasonal dynamics of Actinobacteria populations in four lakes in northeastern Germany. Appl Environ Microbiol. (2006) 72:3489–97. 10.1128/AEM.72.5.3489-3497.2006 16672495 PMC1472390

[38] Eiler A Bertilsson S. Composition of freshwater bacterial communities associated with cyanobacterial blooms in four Swedish lakes. Environ Microbiol. (2004) 6:1228–43. 10.1111/j.1462-2920.2004.00657.x 15560821

[39] Van der Gucht K Vandekerckhove T Vloemans N Cousin S Muylaert K Sabbe K. Characterization of bacterial communities in four freshwater lakes differing in nutrient load and food web structure. FEMS Microbiol Ecol. (2005) 53:205–20. 10.1016/j.femsec.2004.12.006 16329941

[40] Carrillo M Estrada E Hazen TC. Survival and enumeration of the fecal indicators Bifidobacterium adolescentis and *Escherichia coli* in a tropical rain forest watershed. Appl Environ Microbiol. (1985) 50:468–76. 3901921 10.1128/aem.50.2.468-476.1985 PMC238644

[41] Hossain MA Albert MJ Hasan KZ. Epidemiology of shigellosis in Teknaf, a coastal area of Bangladesh: a 10-year survey. Epidemiol Infect. (1990) 105:41–9. 10.1017/S0950268800047622 2200700 PMC2271791

[42] Byappanahalli MN Whitman RL Shively DA Sadowsky MJ Ishii S. Population structure, persistence, and seasonality of autochthonous *Escherichia coli* in temperate, coastal forest soil from a Great Lakes watershed. Environ Microbiol. (2006) 8:504–13. 10.1111/j.1462-2920.2005.00916.x 16478456

[43] Ishii S Yan T Shively DA Byappanahalli MN Whitman RL Sadowsky MJ. Cladophora (Chlorophyta) spp. harbor human bacterial pathogens in nearshore water of Lake Michigan. Appl Environ Microbiol. (2006) 72:4545–53. 10.1128/AEM.00131-06 16820442 PMC1489363

[44] Walk ST Alm EW Calhoun LM Mladonicky JM Whittam TS. Genetic diversity and population structure of *Escherichia coli* isolated from freshwater beaches. Environ Microbiol. (2007) 9:2274–88. 10.1111/j.1462-2920.2007.01341.x 17686024

[45] Walk ST Alm EW Gordon DM Ram JL Toranzos GA Tiedje JM. Cryptic lineages of the genus Escherichia. Appl Environ Microbiol. (2009) 75:6534–44. 10.1128/AEM.01262-09 19700542 PMC2765150

[46] Dowd SE Sun Y Secor PR Rhoads DD Wolcott BM James GA. Survey of bacterial diversity in chronic wounds using pyrosequencing, DGGE, and full ribosome shotgun sequencing. BMC Microbiol. (2008) 8:43. 10.1186/1471-2180-8-43 18325110 PMC2289825

[47] Edwards RA Rodriguez-Brito B Wegley L Haynes M Breitbart M Peterson DM. Using pyrosequencing to shed light on deep mine microbial ecology. BMC Genomics. (2006) 7:57. 10.1186/1471-2164-7-57 16549033 PMC1483832

[48] Huber JA Mark Welch DB Morrison HG Huse SM Neal PR Butterfield DA. Microbial population structures in the deep marine biosphere. Science. (2007) 318:97–100. 10.1126/science.1146689 17916733

[49] Keijser BJ Zaura E Huse SM van der Vossen JM Schuren FH Montijn RC. Pyrosequencing analysis of the oral microflora of healthy adults. J Dent Res. (2008) 87:1016–20. 10.1177/154405910808701104 18946007

[50] Kim BS Kim BK Lee JH Kim M Lim YW Chun J. Rapid phylogenetic dissection of prokaryotic community structure in tidal flat using pyrosequencing. J Microbiol. (2008) 46:357–63. 10.1007/s12275-008-0071-9 18758724

[51] Lliros M Inceoglu O Garcia-Armisen T Anzil A Leporcq B Pigneur LM. Bacterial community composition in three freshwater reservoirs of different alkalinity and trophic status. PLoS ONE. (2014) 9:e116145. 10.1371/journal.pone.0116145 25541975 PMC4277477

[52] Roesch LF Fulthorpe RR Riva A Casella G Hadwin AK Kent AD. Pyrosequencing enumerates and contrasts soil microbial diversity. ISME J. (2007) 1:283–90. 10.1038/ismej.2007.53 18043639 PMC2970868

[53] Qadri F Das SK Faruque AS Fuchs GJ Albert MJ Sack RB. Prevalence of toxin types and colonization factors in enterotoxigenic *Escherichia coli* isolated during a 2-year period from diarrheal patients in Bangladesh. J Clin Microbiol. (2000) 38:27–31. 10618058 10.1128/jcm.38.1.27-31.2000 PMC86010

[54] Begum YA Baby NI Faruque AS Jahan N Cravioto A Svennerholm AM. Shift in phenotypic characteristics of enterotoxigenic Escherichia coli (ETEC) isolated from diarrheal patients in Bangladesh. PLoS Negl Trop Dis. (2014) 8:e3031. 10.1371/journal.pntd.0003031 25032802 PMC4102457

[55] Ud-Din AI Wahid SU Latif HA Shahnaij M Akter M Azmi IJ. Changing trends in the prevalence of Shigella species: emergence of multi-drug resistant *Shigella sonnei* biotype g in Bangladesh. PLoS ONE. (2013) 8:e82601. 10.1371/journal.pone.0082601 24367527 PMC3867351

[56] Qadri F Svennerholm AM Faruque AS Sack RB. Enterotoxigenic Escherichia coli in developing countries: epidemiology, microbiology, clinical features, treatment, and prevention. Clin Microbiol Rev. (2005) 18:465–83. 10.1128/CMR.18.3.465-483.2005 16020685 PMC1195967

[57] Gorra R Webster G Martin M Celi L Mapelli F Weightman AJ. Dynamic microbial community associated with iron-arsenic co-precipitation products from a groundwater storage system in Bangladesh. Microb Ecol. (2012) 64:171–86. 10.1007/s00248-012-0014-1 22349905

[58] Hasan NA Chowdhury WB Rahim N Sultana M Shabnam SA Mai V Metagenomic 16S rDNA Targeted PCR-DGGE in determining bacterial diversity in aquatic ecosystem. Bangladesh Journal Microbiol. (2010) 27:46–50. 10.3329/bjm.v27i2.9171

