# Supplementary material for: Bacterial Community Profiling of Tropical Freshwaters in Bangladesh
Source: Front Public Health. 2019 May 31;7:115. doi: 10.3389/fpubh.2019.00115 (PMC6554655; doi:10.3389/fpubh.2019.00115)
Supplement: Supplementary file 1 [file Data_Sheet_1.pdf]

<u>Supplimentary material</u>

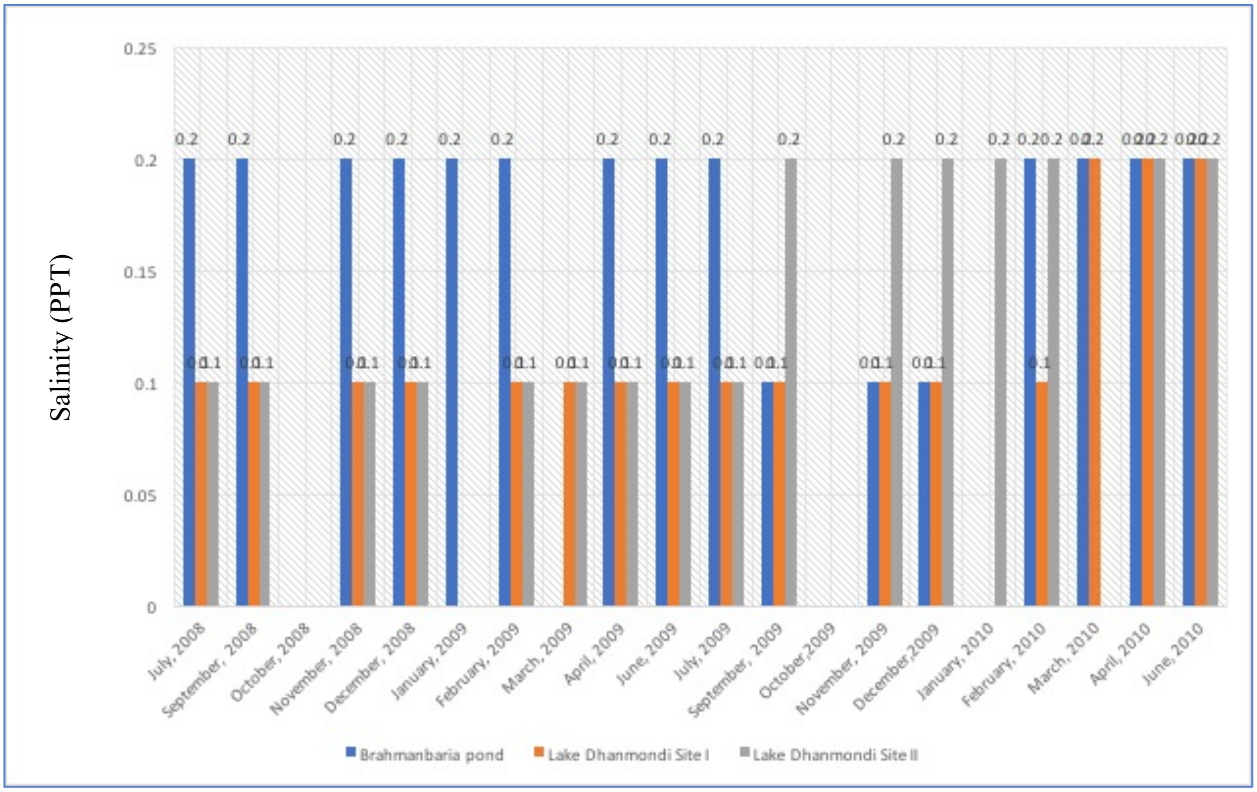

Figure S1: Salinity measured in July 2008 – June 2010 in three sites selected for bacterial community sturcture analysis

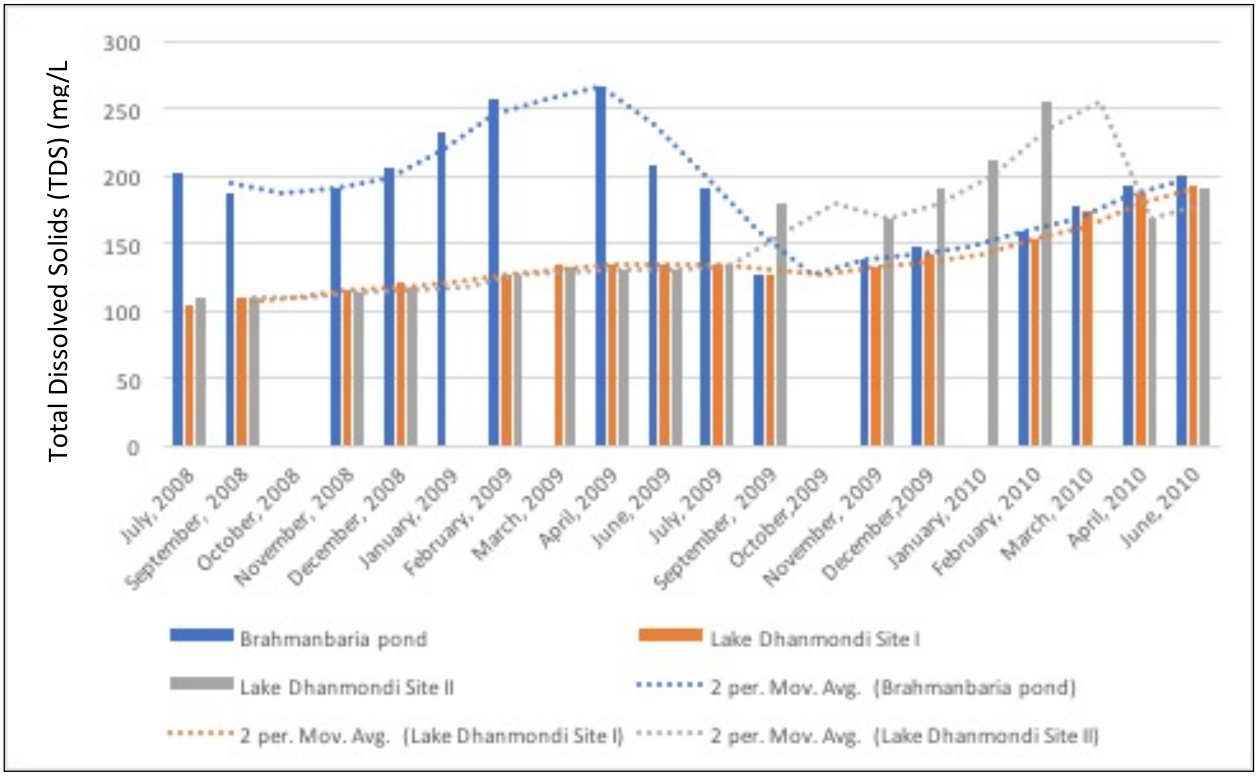

Figure S2: TDS measured in July 2008 – June 2010 in three sites selected for bacterial community sturcture analysis

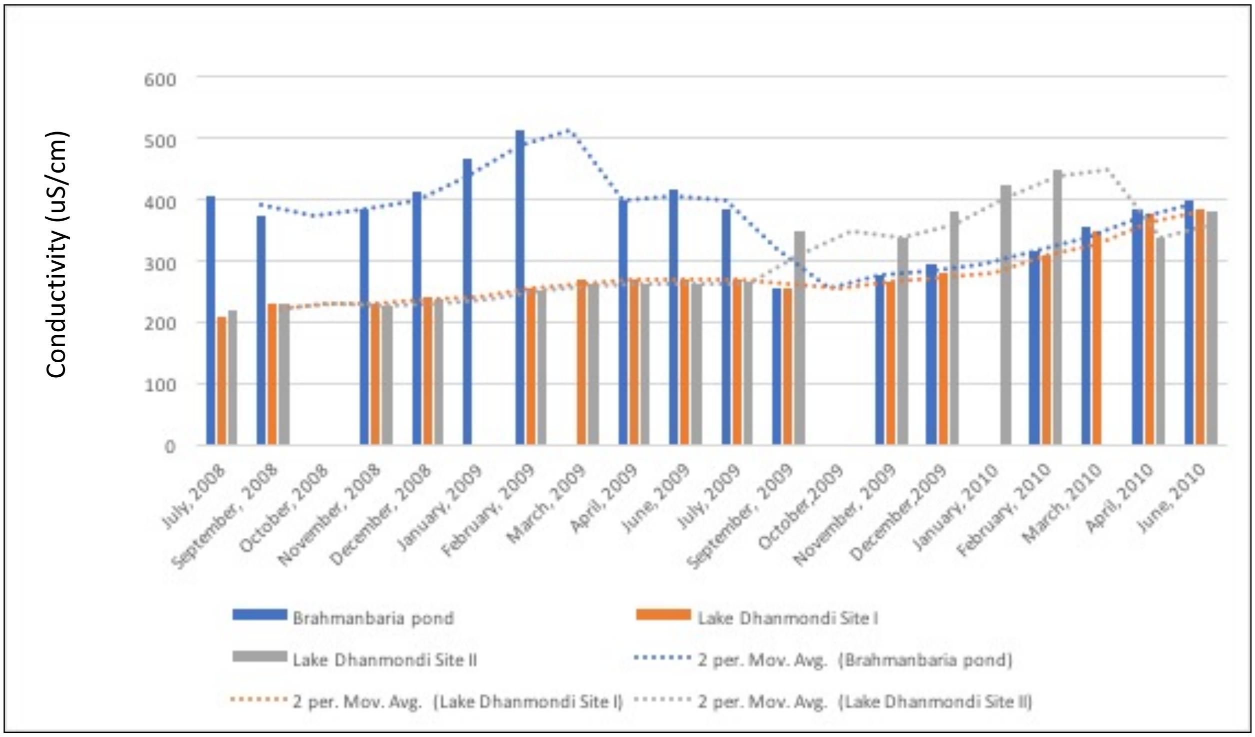

Figure S3: Conductivity measured in July 2008 – June 2010 in three sites selected for bacterial community sturcture analysis

Table S1: Primer sequences of the 9 target genes for multiplex PCR assay

Multiplex PCR sets	Primers	Target Genes	Primer sequence	Amplicon Size (bp)	References
**Set 1**	**ST**	***estA***	5′-GCTAAACCAGTAGAGGTCTTCAAAA-3′<br>5′-CCCGGTACAGAGCAGGATTACAACA-3′	147	1
	**SHIG**	***ial***	5′-CTGGTAGGTATGGTGAGG-3′<br>5′-CCAGGCCAACAATTATTTCC-3′	320	2
	**bfpA**	***bfpA***	5′-TTCTTGGTGCTTGCGTGTCTTTT-3′<br>5′-TTTTGTTTGTTGTATCTTTGTAA-3′	367	3
	**EA**	**pCVD**	5′-CTGGCGAAAGACTGTATCAT-3′<br>5′-CAATGTATAGAAATCCGCTGTT-3′	630	4
**Set 2**	**VT1**	***vt1***	5′-GAAGAGTCCGTGGGATTACG-3′<br>5′-AGCGATGCAGCTATTAATAA-3′	130	5
	**VT2**	***vt2***	5′-ACCGTTTTTCAGATTTTGACACATA-3′<br>5′-TACACAGGAGCAGTTTCAGACAGT-3′	298	6
	**eae**	***eaeA***	5′-CACACGAATAAACTGACTAAAATG-3′<br>5′-AAAAACGCTGACCCGCACCTAAAT-3′	376	7
**Set 3**	**ipaH**	***ipaH***	5′-GCTGGAAAAACTCAGTGCCT-3′<br>5′-CCAGTCCGTAAATTCATTCT-3′	423	8
	**LT**	***eltB***	5′-TCTCTATGTGCATACGGAGC-3′<br>5′-CCATACTGATTGCCGCAAT-3′	322	9

1.Moseley SL, et al. Isolation and nucleotide sequence determination of a gene encoding a heat-stable enterotoxin of Escherichia coli. Infection and Immunity 1983;39: 1167–1174.
2. Frankel G, et al. Detection of Shigella in feces using DNA amplification. Journal of Infectious Diseases 1990; 161: 1252–1256.
3. Yatsuyanagi J, et al. Characterization of enteropathogenic and enteroaggregative Escherichia coli isolated from diarrheal outbreaks. Journal of Clinical Microbiology 2002; 40: 294–297.
4. Pereira AL, et al. Enteroaggregative Escherichia coli virulence markers: positive association with distinct clinical characteristics and segregation into 3 enteropathogenic E. coli serogroups. Journal of Infectious Diseases 2007; 195: 366–374.
5. Pollard DR, et al. Rapid and specific detection of verotoxin genes in Escherichia coli by the polymerase chain reaction. Journal of Clinical Microbiology 1990; 28: 540–545.
6. Lindqvist R. Preparation of PCR samples from food by a rapid and simple centrifugation technique evaluated by detection of Escherichia coli O157:H7. International Journal of Food Microbiology 1997; 37: 73–82.
7. Svenungsson B, et al. Enteropathogens in adult patients with diarrhea and healthy control subjects: a 1-year prospective study in a Swedish clinic for infectious diseases. Clinical Infectious Diseases 2000; 30: 770–778.
8. Tornieporth NG, et al. Differentiation of pathogenic Escherichia coli strains in Brazilian children by PCR. Journal Clinical Microbiology 1995; 33: 1371–1374.
9. Inoue T, et al. Amino acid sequence of heat-labile enterotoxin from chicken enterotoxigenic Escherichia coli is identical to that of human strain H 10407. FEMS Microbiology Letters 1993; 108: 157–161.

Table S2: Identification of DGGE bands excised for sequencing from Brahmanbaria pond in July 2008-July 2009

Band No.	Closest relative	Maximum identity with closest relative (%)	GenBank Accession Number
A1	*Acinetobacter* sp. PHLE-7	99	MK271399
A2	*Escherichia coli* O7:K1 str. CE10	93	MK271400
A3	*Escherichia coli* O7:K1 str. CE10	99	MK271401
A4	*Aeromonas caviae* strain: JCM 1060	99	MK271402
A5	*Aeromonas hydrophila* strain: NBRC 12658	100	MK271403
A6	*Acinetobacter* sp. PHLE-7	99	MK271404
A7	*Comamonas aquatica* strain: NBRC 14918	99	MK271405
A8	*Kurthia* sp. VITA1	90	MK271406
A9	*Kurthia* sp. VITA1	96	MK271407
A10	*Kurthia* sp. VITA1	100	MK271408
A11	*Aeromonas* sp. PC IW 14	94	MK271409
A12	*Aeromonas caviae* strain: JCM 1060	99	MK271410
A13	*Cyanobium* sp. JJM10D4	99	MK271411
A14	*Cyanobium* sp. JJ9-A3	99	MK271412
A15	*Shigella sonnei* 53G<br>*Escherichia coli* clone:1C4	99	MK271413
A16	*Aeromonas caviae* strain: JCM 1060	100	MK271414
A17	*Aeromonas caviae* strain: JCM 1060	100	MK271415
A18	*Escherichia coli* O7:K1 str. CE10	96	MK271416
A19	*Comamonas aquatica* strain: NBRC 14918	99	MK271417
B20	*Cyanobium* sp. JJM10D4	99	MK271418
B21	*Acinetobacter johnsonii* strain MA19	93	MK271419
B22	*Acinetobacter* sp. STM18	100	MK271420

Table S2: Continued.

Band No.	Closest relative	Maximum identity with closest relative (%)	GenBank Accession Number
B23	*Comamonas* sp. GPTSA28	100	MK271421
B25	*Comamonas aquatica* strain: NBRC 14918	97	MK271422
B26	*Comamonas* sp. 6.6	99	MK271423
B27	*Comamonas* sp. GAH8	100	MK271424
B28	*Comamonas* sp. LR38	87	MK271425
B29	*Aeromonas caviae* strain: JCM 1060	99	MK271426
B30	*Aeromonas veronii* strain IH101	100	MK271427
B31	*Cyanobium* sp.	99	MK271428
B32	*Cyanobium* sp. JJM10D5	99	MK271429
B33	*Cyanobium* sp. JJNV	99	MK271430
B34	*Kurthia gibsonii* strain AU23	96	MK271431
B35	*Kurthia gibsonii* strain AU23	100	MK271432
B36	*Exiguobacterium* sp. REC21	99	MK271433
B37	*Exiguobacterium indicum* strain: HHS 31	99	MK271434
B38	*Cyanobium* sp. JJNV	99	MK271435
B39	*Aeromonas caviae* strain UMS20/10	99	MK271436
B40	*Aeromonas caviae* strain: JCM 1060	100	MK271437
B41	*Comamonas kersterii* strain LMG 5323	94	MK271438
B42	*Bacillus* sp. NBRC 101285	98	MK271439
B43	*Comamonas kersterii* strain LMG 5323	99	MK271440
B44	*Comamonas aquatica* strain: NBRC 14918	97	MK271441
B46	*Microcystis aeruginosa* strain SPC 777	99	MK271442
B47	*Vogesella* sp. TPS6	99	MK271443

Table S2: Continued.

Band No.	Closest relative	Maximum identity with closest relative (%)	GenBank Accession Number
B48	*Vogesella perlucida* strain GR-9	99	MK271444
B49	*Vogesella* sp. 3F	90	MK271445
B50	*Vogesella* sp. AKB-2008-TE15	88	MK271446
B51	*Vogesella* sp. TPS6	98	MK271447
B52	*Microcystis aeruginosa* PCC7005	91	MK271448
B53	*Vogesella* sp. TPS6	99	MK271449

Table S3: Identification of DGGE bands excised for sequencing from Dhanmondi Lake site I in July 2008-July 2009

Band No.	Closest relative	Maximum identity with closest relative (%)	GenBank Accession Number
C1	*Cyanobium* sp. JJ9-A3	96	MK271450
C2	*Candidatus Pelagibacter* sp. IMCC9063	91	MK271451
C3	*Vogesella perlucida* strain GR-9	99	MK271452
C4	*Vogesella perlucida* strain GR-9	98	MK271453
C5	*Aeromonas caviae* strain UMS20/10	100	MK271454
C6	*Pseudomonas* sp. 3CB6	82	MK271455
C7	*Escherichia coli* P12b	98	MK271456
C8	*Escherichia coli* strain B_IV_1E47	100	MK271457
C9	*Acinetobacter* sp. AUH-JLM210	85	MK271458
C10	*Vogesella perlucida*	98	MK271459
C11	*Aeromonas* sp. L57	99	MK271460
C12	*Aeromonas veronii* strain:N35	98	MK271461
C13	*Methylophilus* sp. CBMB161	99	MK271462
C14	*Burkholderiales* sp. RCPCd10	97	MK271463
C15	*Aeromonas caviae* strain UMS20/10	95	MK271464
C16	*Comamonas* sp. GPTSA28	99	MK271465
C17	*Comamonas* sp. GPTSA28	90	MK271466
C18	*Comamonas* sp. GPTSA28	99	MK271467
C19	*Comamonas* sp. GPTSA28	99	MK271468
C20	*Enterobacter* sp. 7J2	85	MK271469
C21	*Comamonas testosteroni* strain: NBRC 12048	98	MK271470
C22	*Kurthia* sp. PAOGL173	97	MK271471
C23	*Kurthia gibsonii* strain J9B-74	99	MK271472
C24	*Kurthia* sp. PAOGL173	99	MK271473

C25	*Aeromonas* sp. PC IW 14	98	MK271474
C26	*Aeromonas caviae* strain: JCM 1060	99	MK271475
D27	*Comamonas* sp. SJM	100	MK271476
D28	*Bacillus* sp. NBRC 101285	94	MK271477
D29	*Cyanobium* sp. LB03	98	MK271478
D30	*Bacillus* sp. SW-PTK	99	MK271479
D31	*Bacillus anthracis* strain FCC158	99	MK271480
D32	*Bacillus anthracis* strain FCC158	99	MK271481
D33	*Bacillus anthracis* strain FCC158	99	MK271482
D34	*Shigella flexneri* 2a str. 301/ *Escherichia coli* strain P33	95	MK271483
D35	*Comamonas* sp. SJM	99	MK271484
D36	*Aeromonas veronii* strain DF201101	99	MK271485
D37	*Vogesella* strain GR-9	99	MK271486
D38	*Vogesella* sp. AKB-2008-TE22	97	MK271487
D39	*Vogesella* sp. AKB-2008-TE22	95	MK271488
D40	*Vogesella* perlucida strain GR-9	99	MK271489
D41	*Vogesella* sp. AKB-2008-TE22	96	MK271490
D42	*Aeromonas jandaei* strain HX201006-2	87	MK271491
D43	*Aeromonas jandaei* strain HX201006-2	95	MK271492
D44	*Bacillus* sp. SW-PTK	99	MK271493
D45	*Cyanobium* sp. LB03	98	MK271494
D47	*Polynucleobacter cosmopolitanus* strain MWH-Braz-FAM2H	95	MK271495
D48	*Cyanobium* sp. JJ9-A3 strain JJ9-A3	97	MK271496
D49	*Cyanobium* sp. JJ9-A3 strain JJ9-A3	98	MK271497
D50	*Bacillus anthracis* strain FCC158	100	MK271498
D51	*Aeromonas caviae* strain UMS20/10	99	MK271499

Table S3: Continued.

Band No.	Closest relative	Maximum identity (%)	GenBank Accession Number
D52	*Aeromonas* sp. LH2	100	MK271500
D53	*Lysinibacillus* sp. PRM9	99	MK271501
D54	*Escherichia coli* strain 123	100	MK271502
D55	*Comamonas aquatica* strain: NBRC 14918	99	MK271503
D56	*Exiguobacterium acetylicum* strain W5	100	MK271504
D57	*Vogesella* sp. AKB-2008-TE22	99	MK271505
D58	*Vogesella perlucida* strain GR-9	97	MK271506
E60	*Acinetobacter junii* strain NB5_3B	94	MK271507
E61	*Acinetobacter* sp. strain: M145	96	MK271508
E62	*Acinetobacter* sp. M114	98	MK271509
E63	*Comamonas* sp. GPTSA28	99	MK271510
E64	*Vogesella perlucida* strain GR-9	94	MK271511
E65	*Bacillus anthracis* strain FCC158	99	MK271512
E66	*Vogesella perlucida* strain GR-9	99	MK271513
E67	*Cyanobium* sp. JJNV strain JJNV	92	MK271514
E68	*Cyanobium* sp. JJ9-A3 strain JJ9-A3	97	MK271515
E69	*Cyanobium* sp. JJ9-A3 strain JJ9-A3	94	MK271516
E70	*Aeromonas enteropelogenes* strain AIMST Ehe25	100	MK271517
E71	*Aeromonas caviae* strain: JCM 1060	99	MK271518
E72	*Aeromonas enteropelogenes* strain AIMST Ehe25	99	MK271519
E73	*Bacillus anthracis* strain FCC158	99	MK271520
E74	*Vogesella perlucida* strain GR-9	98	MK271521
E75	*Vogesella* sp. AKB-2008-TE22	92	MK271522
E76	*Comamonas* sp. GPTSA28	99	MK271523

Table S3: Continued.

Bnad No.	Closest relative	Maximum identity with closest relative (%)	GenBank Accession Number
E77	*Comamonas kersterii* strain LMG 5323	94	MK271524
E78	*Vogesella* sp. TPS6	98	MK271525
E79	*Vogesella* sp. AKB-2008-TE22	99	MK271526
E80	*Vogesella* sp. TPS6	99	MK271527
E81	*Comamonas* sp. 6.6	90	MK271528
E82	*Comamonas* sp. GAH8 strain GAH8	99	MK271529
E83	*Comamonas* sp. 6.6	99	MK271530
E84	*Comamonas kersterii* strain LMG 5323	86	MK271531
E85	*Comamonas* sp. 6.6	97	MK271532
E86	*Comamonas* sp. GAH8 strain GAH8	92	MK271533
E87	*Comamonas* sp. 6.6	99	MK271534
E88	*Comamonas* sp. GAH8	99	MK271535
E89	*Vogesella* sp. strain AKB-2008-TE22	94	MK271536
E90	*Vogesella* sp. TPS6	99	MK271537
E91	*Vogesella* sp. strain AKB-2008-TE22	90	MK271538
E92	*Vogesella* sp. 389	97	MK271539
E93	*Candidatus Pelagibacter* sp. IMCC9063	90	MK271540
E94	*Cyanobium* sp.	99	MK271541
E95	*Cyanobium* sp. strain JJNV	98	MK271542
E96	*Vogesella perlucida* strain GR-9	99	MK271543
E97	*Vogesella perlucida* strain GR-9	99	MK271544
E98	*Vogesella perlucida* strain GR-9	99	MK271545
E99	*Vogesella* sp. strain AKB-2008-TE22	99	MK271546

Table S3: Continued.

Band No.	Closest relative	Maximum identity with closest relative (%)	GenBank Accession Number
E100	*Vogesella* sp. strain AKB-2008-TE22	98	MK271547
E101	*Vogesella* sp. TPS6	99	MK271548
E103	*Bacillus* sp. NBRC 101285	100	MK271549
E104	*Pseudomonas otitidis* strain R6-410	97	MK271550
E105	*Plesiomonas shigelloides* (ATCC 14029T)	99	MK271551
E106	*Plesiomonas shigelloides* (ATCC 14029T)	100	MK271552
E107	*Acinetobacter baumannii* Naval-17 clone 1122233346963	98	MK271553
E108	*Vogesella* sp. strain AKB-2008-TE22	98	MK271554
E109	*Vogesella* sp. TPS6	98	MK271555

Table S4: Identification of the DGGE bands excised for sequencing from Dhanmondi Lake site II in July 2008 to July 2009

Band No.	Closest relative	Maximum Identity with closest relative (%)	GenBank Accession Number
F1	*Aeromonas* sp. SW42	99	MK271556
F2	*Aeromonas* sp. B1RO11	98	MK271557
F3	*Aeromonas punctata* strain RCPS-2	99	MK271558
F4	*Vogesella* sp. AKB-2008-TE22	87	MK271559
F5	*Candidatus Pelagibacter* sp. IMCC9063	92	MK271560
F6	*Comamonas* sp. GPTSA28	99	MK271561
F7	*Cyanobium* sp. JJ9-A3	98	MK271562
F8	*Aquabacterium* sp.	93	MK271563
F9	*Cyanobium* sp. LB03	96	MK271564
F10	*Comamonas* sp. GPTSA28	97	MK271565
F11	*Comamonas* sp. GPTSA28	99	MK271566
F12	*Bacterium* Ellin7531	94	MK271567
F13	*Methylobacter* sp. HG-1	97	MK271568
F14	*Methyloversatilis universalis*	95	MK271569
F15	*Panacagrimonas perspica*	96	MK271570
F16	*Shigella flexneri* strain MP12_1A<br>*Escherichia coli* strain HG9_1A	100	MK271571
F17	*Vogesella indigofera* strain BPB-P	94	MK271572
F18	*Comamonas* sp. GPTSA28	99	MK271573
F19	*Comamonas* sp. GPTSA28	99	MK271574
F20	*Comamonas* sp. GPTSA28	99	MK271575
F21	*Comamonas kersterii*	98	MK271576

Table S4: Continued.

Band No.	Closest relative	Maximum Identity with closest relative (%)	GenBank Accession Number
F22	*Comamonas* sp. GPTSA28	99	MK271577
F23	*Comamonas* sp. GPTSA28	97	MK271578
F24	*Enterobacter* sp. 9B_2	82	MK271579
F25	*Comamonas* sp. GPTSA28	99	MK271580
F26	*Candidatus Pelagibacter* sp. IMCC9063	92	MK271581
F27	*Exiguobacterium indicum*	99	MK271582
F28	*Cyanobium* sp. LB03	94	MK271583
F29	*Escherichia coli* SE15<br>*Shigella flexneri* strain MP12_1A	94	MK271584
F30	*Plesiomonas* sp. F54	96	MK271585
F31	*Vogesella* sp. TPS6	99	MK271586
F33	*Cyanobium* sp. JJM10D5	92	MK271587
F34	*Aeromonas punctata* strain RCPS-	99	MK271588
F35	*Aeromonas hydrophila* strain AIMST	98	MK271589
F36	*Aeromonas veronii* strain CYJ209	98	MK271590
F37	*Vogesella* sp. AKB-2008-TE22	98	MK271591
F38	*Vogesella* sp. TPS6	99	MK271592
F39	*Aeromonas hydrophila* strain Tw2	99	MK271593
F40	*Plesiomonas shigelloides* strain DSM 8224	100	MK271594
F41	*Aeromonas hydrophila* subsp. Ranae	95	MK271595
F42	*Vogesella* sp. AKB-2008-TE22	98	MK271596
F43	*Vogesella* sp. TPS6	98	MK271597

Table S4: Continued.

Band No.	Closest relative	Maximum Identity with closest relative (%)	GenBank Accession Number
F44	*Aeromonas hydrophila*	99	MK271598
G46	*Kurthia gibsonii* strain AU23	97	MK271599
G47	*Bacillus cereus* strain YC-16	100	MK271600
G48	*Bacillus cereus* strain YC-16	100	MK271601
G49	*Aeromonas aquariorum* strain N2	97	MK271602
G50	*Aeromonas hydrophila* strain 5_3C	97	MK271603
G51	*Vogesella* sp. AKB-2008-TE22	98	MK271604
G52	*Bacillus* sp. NRS-810	87	MK271605
G53	*Vogesella* sp. TPS6	98	MK271606
G54	*Vogesella* sp. TPS6	98	MK271607
G55	*Vogesella* sp. TPS6	99	MK271608
G57	*Cyanobium* sp. JJ9-A3	98	MK271609
G58	*Cyanobium* sp. LB03	97	MK271610
G59	*Aeromonas caviae*	99	MK271611
G60	*Comamonas* sp. GPTSA28	100	MK271612
G61	*Comamonas kersterii*	98	MK271613
G62	*Polynucleobacter cosmopolitanus*	99	MK271614
G63	*Cyanobium* sp. JJNV	99	MK271615
G65	*Comamonas kersterii*	98	MK271616
G66	*Vogesella* sp. TPS6	98	MK271617
G67	*Cyanobium* sp. JJNV	99	MK271618
G68	*Cyanobium* sp. LB03	99	MK271619

Table S4: Continued...

Band No.	Closest relative	Maximum Identity with closest relative (%)	GenBank Accession Number
G69	*Aeromonas caviae*	99	MK271620
G70	*Aeromonas caviae*	98	MK271621
G71	*Aeromonas* sp.	99	MK271622
G72	*Comamonas kersterii*	98	MK271623
G73	*Comamonas kersterii*	98	MK271624
G74	*Comamonas kersterii*	98	MK271625
G75	*Comamonas kersterii*	98	MK271626
G76	*Lysinibacillus sphaericus* strain VCRC B547	99	MK271627
G77	*Vogesella* sp. TPS6	98	MK271628
G78	*Vogesella* sp. TPS6	90	MK271629
G79	*Vogesella* sp. 3F	95	MK271630
G80	*Cyanobium* sp. JJ9-A3	98	MK271631
G81	*Vogesella* sp. TPS6	95	MK271632

Table S5: Distribution of virulence genes of *E. coli* and *Shigella* spp. in differenrent months in three sampling sites

Sampling sites	Year	Months									
		**Jul.**	**Sept.**	**Oct.**	**Nov.**	**Dec.**	**Jan**	**Feb.**	**Mar.**	**Apr.**	**Jun.**
**Brahmanbaria Pond**	2008-2009	*eae,* pCVD, *ipaH*	*eae, eltB*	ND	*eae, eltB ipaH*		*eae*		ND	*vt1* + *eae*	*eae*
	2009-2010	*eae*	*eltB*	ND	*vt2* + *eae ipaH*		*ipaH*		ND		
**Lake Dhanmondi site I, Dhaka**	2008-2009			ND	*ipaH*	*eltB*	ND			*eae*	
	2009-2010			ND	*eae*		ND	*eltB* + *estA, ipaH*		*eae*	*eae*
**Lake Dhanmondi site II, Dhaka**	2008-2009	*eae eltB* + *estA*		ND	*eae*		ND				*eae*
	2009-2010	*estA*		ND		*eltB*	ND		*eae*	*eae, eltB*	*eae*

** Note: EPEC= *eae/eae+bfp*, ETEC= *eltB/estA/eltB+estA*, EAEC= pCVD, EIEC/ *Shigella = ial/ipaH/ial+ipaH,* EHEC= *vt1/vt1+eae/vt2/vt2+eae/ vt1+vt2+eae*

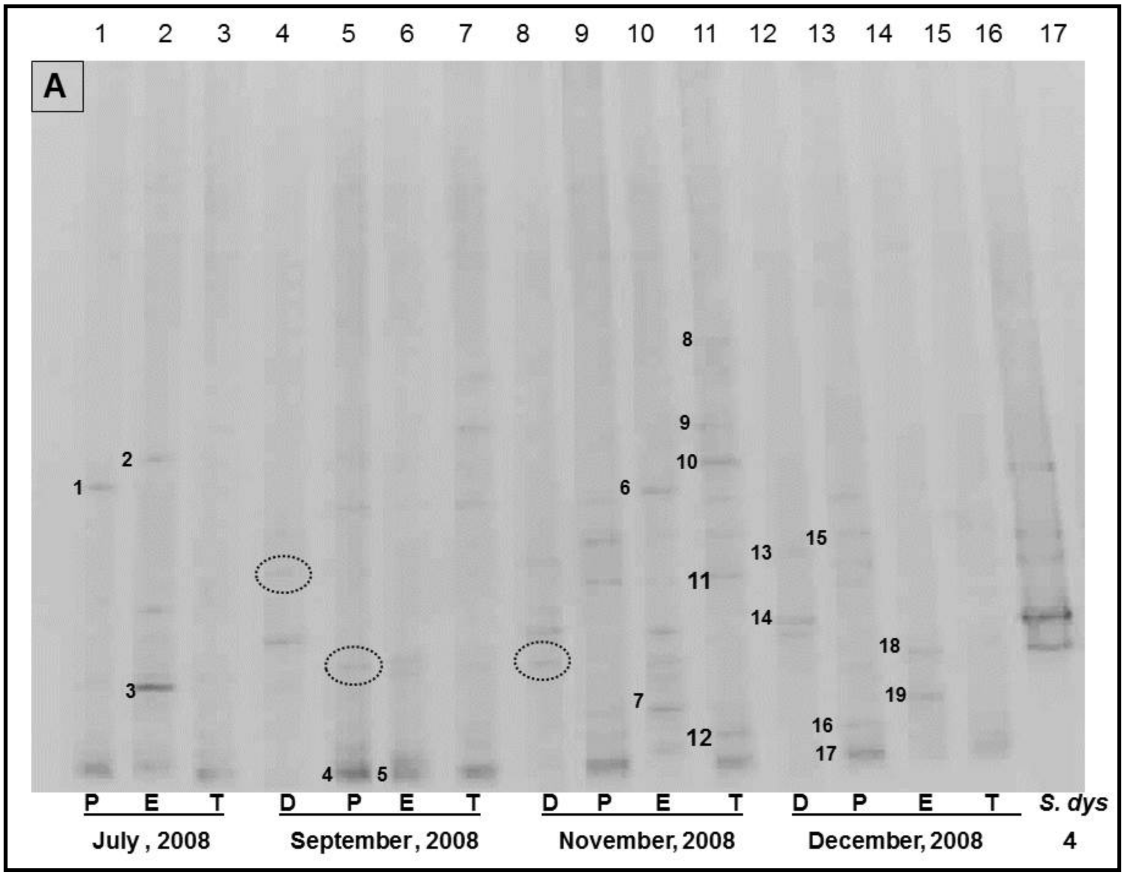

Figure S4: The representative DGGE profiles of bacterial community of Brahmanbaria pond in July, 2008 - December, 2008. D, P, E and T stand for the Direct, Pre-enriched, Enriched and Total heterotrophic DNA samples

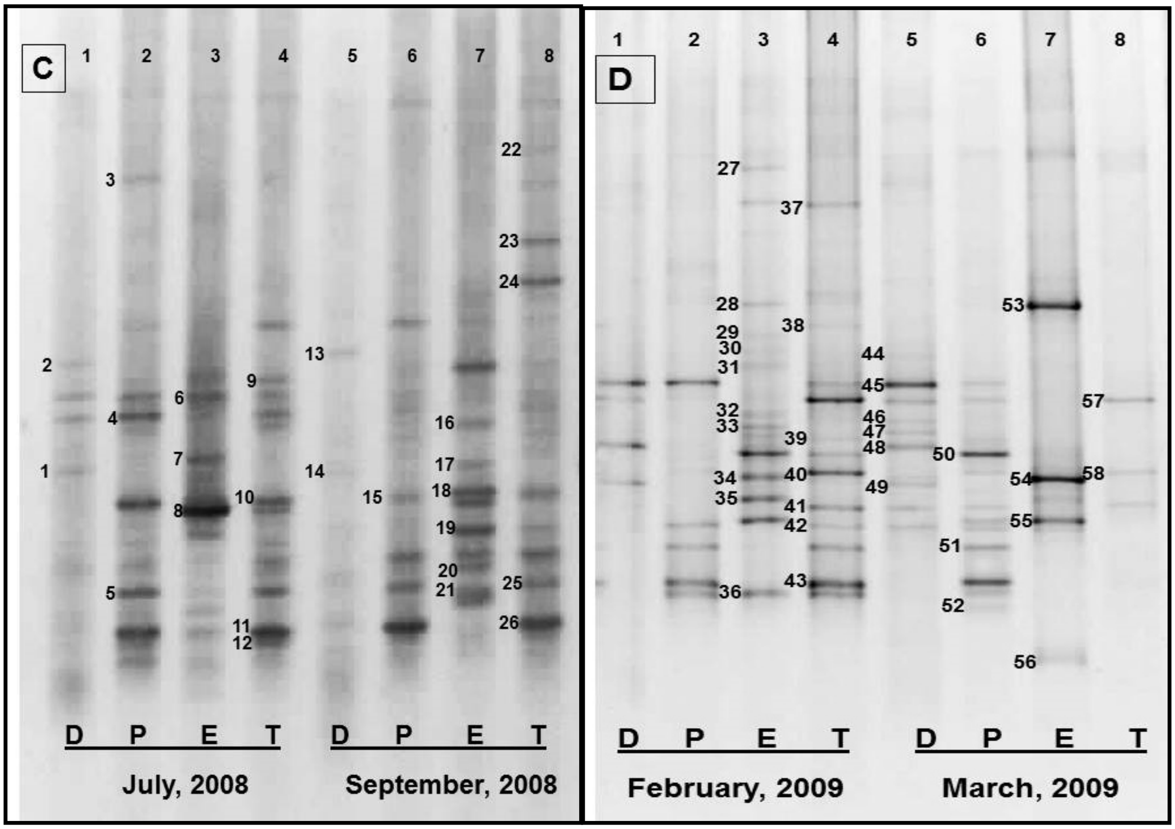

Figure S5: The representative DGGE profiles (Figs C & D) of bacterial community of Dhanmondi Lake site I in July 2008 - March 2009. D, P, E and T stand for the Direct, Pre-enriched, Enriched and Total heterotrophic DNA samples

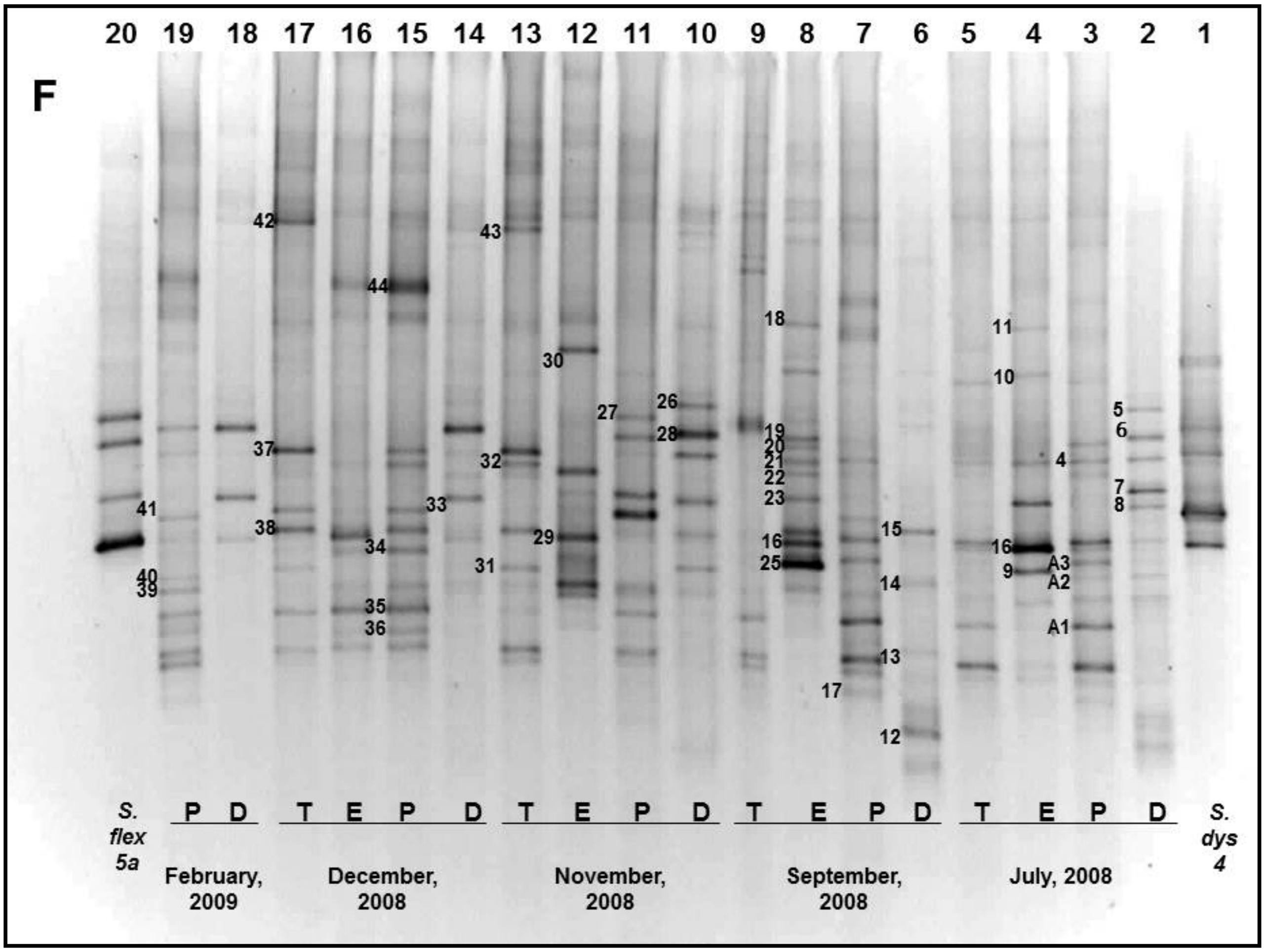

Figure S6: The representative DGGE profiles of bacterial community of Dhanmondi Lake site II in July 2008 - February 2009. Lane 1 and 20 represent the DNA samples of *Shigella* spp. used as positive controls. D, P, E and T stand for the Direct, Pre-enriched, Enriched and Total heterotrophic DNA samples

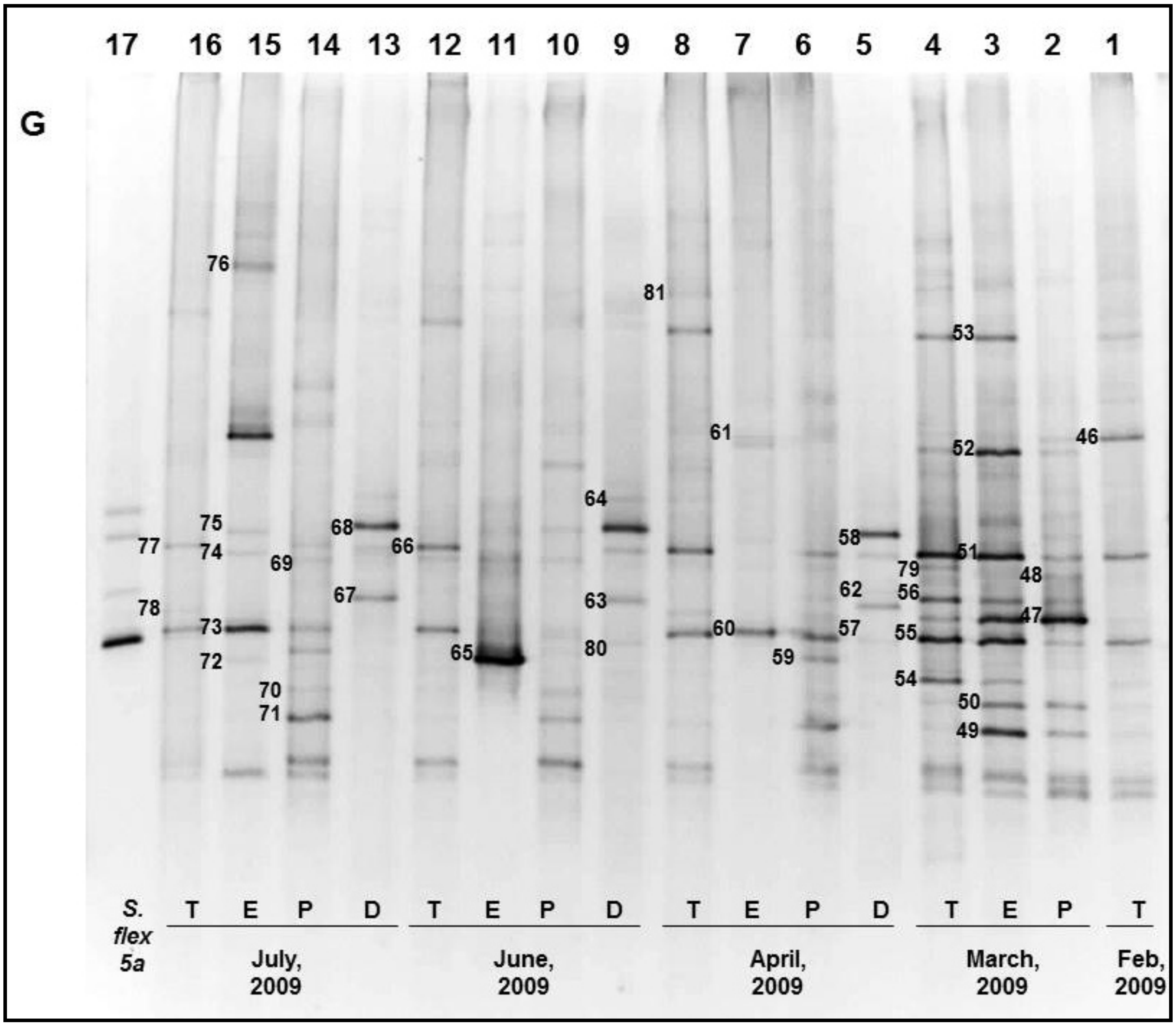

Figure S7: The representative DGGE profiles of bacterial community of Dhanmondi Lake site II in February 2009 - July 2009. Lane 19 represents the DNA samples of *Shigella* spp. used as positive controls. D, P, E and T stand for the Direct, Pre-enriched, Enriched and Total heterotrophic DNA samples
